# Gate-tuned graphene meta-devices for dynamically controlling terahertz wavefronts

**DOI:** 10.1515/nanoph-2021-0801

**Published:** 2022-03-31

**Authors:** Qiushi Li, Xiaodong Cai, Tong Liu, Min Jia, Qiong Wu, Haoyang Zhou, Huanhuan Liu, Qianqian Wang, Xiaohui Ling, Cong Chen, Fan Ding, Qiong He, Yuanbo Zhang, Shiyi Xiao, Lei Zhou

**Affiliations:** Key Laboratory of Specialty Fiber Optics and Optical Access Networks, Joint International Research Laboratory of Specialty Fiber Optics and Advanced Communication, Shanghai Institute for Advanced Communication and Data Science, Shanghai University, Shanghai 200444, China; State Key Laboratory of Surface Physics, Key Laboratory of Micro and Nano Photonic Structures (Ministry of Education) and Physics Department, Fudan University, Shanghai 200433, China; Department of Electrical and Electronic Engineering, Southern University of Science and Technology, Shenzhen 518055, China; College of Physics and Electronic Engineering, Hengyang Normal University, Hengyang 421002, China; School of Electronic Information, Wuhan University, Wuhan 430072, China; China Ship Development and Design Center, Wuhan 430064, China; Fudan University, Academy for Engineering and Technology, Shanghai 200433, China; Collaborative Innovation Centre of Advanced Microstructures, Nanjing 210093, China

**Keywords:** coupled mode theory, graphene, metasurfaces, terahertz, wavefront manipulations

## Abstract

Dynamical controls on terahertz (THz) wavefronts are crucial for many applications, but available mechanism requests tunable elements with sub-micrometer sizes that are difficult to find in the THz regime. Here, different from the *local-tuning* mechanism, we propose an alternative approach to construct wavefront-control meta-devices combining specifically designed metasurfaces and *globally tuned* graphene layers. Coupled-mode-theory (CMT) analyses reveal that graphene serves as a tunable loss to drive the whole meta-device to transit from one functional phase to another passing through an intermediate regime, exhibiting distinct far-field (FF) reflection wavefronts. As a proof of concept, we design/fabricate a graphene meta-device and experimentally demonstrate that it can reflect normally incident THz wave to pre-designed directions with different polarizations under appropriate gating voltages. We finally design a graphene meta-device and numerically demonstrate that it can generate vectorial THz beams with continuously varying polarization distributions upon gating. These findings pave the road to realizing a wide range of THz applications, such as sensing, imaging, and wireless communications.

## 1 Introduction

Dynamically controlling terahertz (THz) wavefronts play a vital role in THz technologies such as sensing, imaging, and wireless communications, which has attracted intensive attention recently. Unfortunately, traditional THz devices are typically bulky in sizes and exhibit limited functionalities, not mentioning the added complications in making the devices actively tunable. The ultimate reasons are the weak interactions between natural materials and the THz waves.

Recently, fast developments on metasurfaces provide new opportunities for achieving dynamic wavefront-controls on electromagnetic (EM) waves. Metasurfaces are ultra-thin metamaterials constructed by subwavelength planar microstructures (e.g., meta-atoms) exhibiting tailored EM responses [1], [2], [3] arranged in certain global sequences. Many fascinating wave-manipulation effects were demonstrated based on metasurfaces, such as anomalous reflection/refraction [4], [5], [6], [7], [8], [9], polarization manipulation [10], [11], [12], [13], photonic spin-Hall effect [14], [15], [16], [17], and meta-hologram [18], [19], [20], [21], [22], [23]. Moreover, integrating active elements (e.g., PIN diodes and varactors) into the metasurface designs, one can realize “tunable” meta-devices capable of dynamically controlling the wavefronts of EM waves reflected by or transmitted through the meta-devices with inserted active elements *individually* biased [24], [25], [26], [27]. Many tunable meta-devices have been successfully constructed in the microwave regime, with wave-manipulation functionalities ranging from beam-steering [28], [29], [30], [31], programmable holograms [32, 33], to dynamic imaging [34, 35]. However, such active meta-devices are difficult to realize at frequencies higher than THz, due to both challenges in finding deep-subwavelength-sized active elements integrated into the THz meta-atoms and the significantly increased difficulties in *individually* tuning these meta-atoms. Instead, most tunable meta-devices realized in the THz regime typically involve “active” layers (e.g., graphene, semi-conductor layers) controlled as a whole. As the result, typically these meta-devices can only control EM waves in a uniform fashion (e.g., amplitude control or polarization control [36], [37], [38]), but cannot control the EM wavefronts dynamically due to deficiencies in modulating EM responses at the deep-subwavelength scales.

In this paper, we propose to build *globally tuned* graphene meta-devices for dynamically tuning THz wavefronts *without* using the local tuning mechanism. Combining gate-controlled graphene and a purposely designed metasurface, we find that the whole device can exhibit different reflection wavefronts with the graphene layer gated uniformly under different voltages. The underlying physics is that the metasurface exhibits multiple functional phases with distinct FF reflection properties, and the graphene serves as a tunable loss to drive the whole device transit between two different functionality phases upon external gating. To illustrate our general strategy, we experimentally demonstrate a gate-controlled meta-deflector that can reflect normally incident THz wave to pre-designed directions with specific polarizations under two particular gating voltages. Furthermore, we can also coherently overlap two FF scattered fields generating new reflection wavefronts, which can be dynamically tuned by external gating. As an illustration, we design a tunable meta-device and numerically demonstrate that it can generate vectorial THz beams with continuously varying polarization distributions, as we change the gating voltage continuously.

## 2 Basic concept

**Figure 1:**
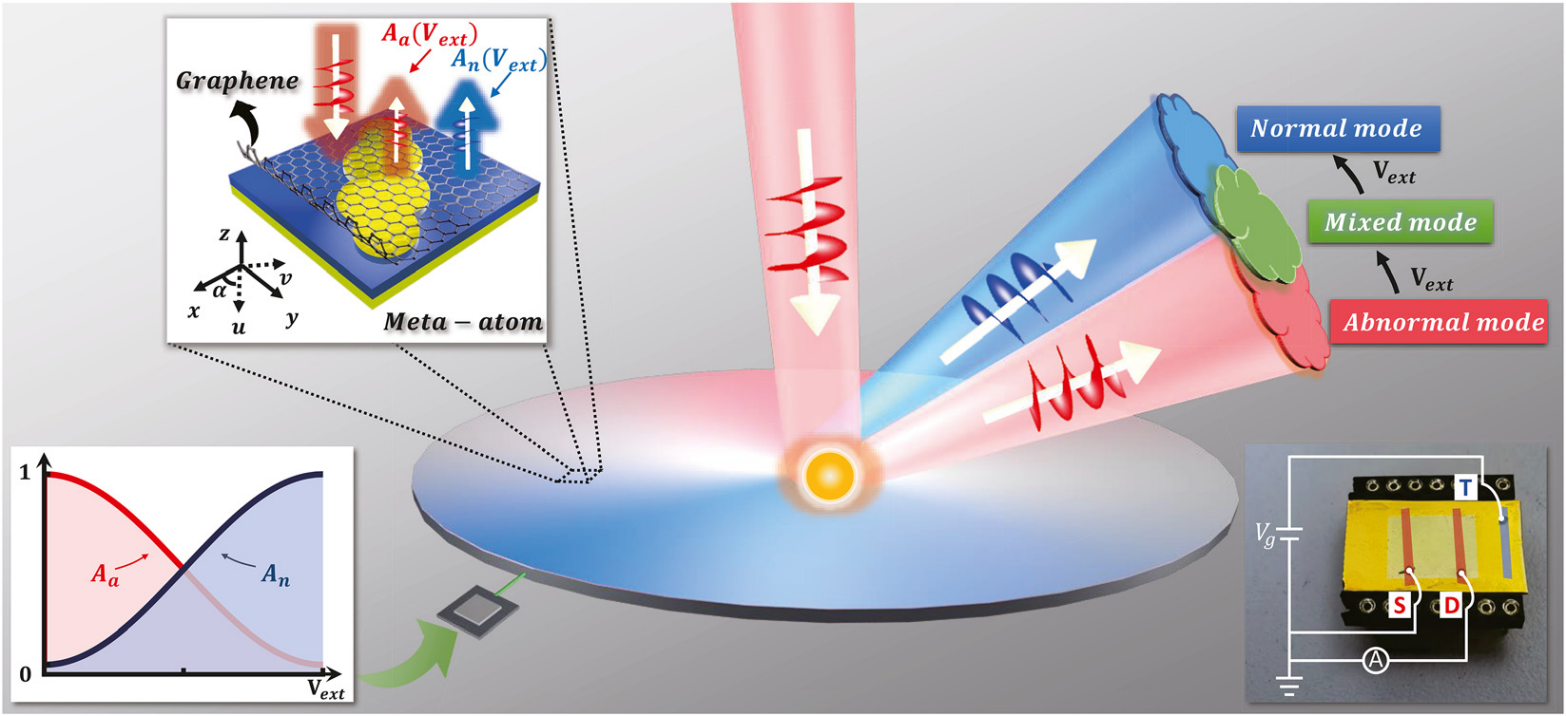
Schematic of *globally tuned* graphene meta-device for dynamic wavefront control. Such meta-device is constructed by a series of identical meta-atoms exhibiting different orientation angles with electrically gated graphene on the top. By varying the external stimuli $V_{\mathrm{ext}}$ (left-bottom inset), electromagnetic responses for all meta-atoms (left-top inset) can be tuned in a same manner and transform the FF scattering modes from one mode (abnormal mode) to another (normal mode). Moreover, when both two FF scattering modes are overlap together, interference between these two beams (a mixed mode) can generate a new reflection wavefront controlled by varying the gating voltage. The right bottom inset shows the photograph of the fabricated meta-device with “T” denoting a bias connected to the ion-gel and “S” and “D” being drain–source base to measure drain–source current.

Figure 1 schematically illustrates the proposed meta-device, which can generate dynamically tunable wavefronts upon global tuning, as illuminated by a circularly-polarized light beam. Such meta-device is constructed by a series of *identical* reflective-type meta-atoms exhibiting orientation angles described by a pre-designed function α(r) [see inset to Figure 1], with an electrically gated graphene put on its top. The constitutional meta-atom is supposed to exhibit a reflection Jones matrix $J=\left(\begin{array}{ll} r_{uu} & 0\\ 0 & r_{vv} \end{array}\right)$ with $\left\{\hat{u},\hat{v}\right\}$ denoting their principal axes, which are rotated by an angle of α(r) for a meta-atom located at position r . Gating the graphene uniformly, $r_{uu}$ and $r_{vv}$ must vary against the gating voltage $V_{\mathrm{ext}}$ , making the local Jones matrix J also a function of $V_{\text{ext}}$ . Then, in laboratory coordinate system $\left\{\hat{x},\hat{y}\right\}$ , the Jones matrix distribution of the whole meta-device, represented in circular polarization (CP) bases, is found as

(1) $$\tilde{J}\left(r;V_{\mathrm{ext}}\right)=S^{-1}\cdot R{\left(r\right)}^{-1}\cdot \left(\begin{array}{ll} r_{uu}\left(V_{\mathrm{ext}}\right) & 0\\ 0 & r_{vv}\left(V_{\mathrm{ext}}\right) \end{array}\right)\cdot R\left(r\right)\cdot S\text{,}$$

where $S=\frac{\sqrt{2}}{2}\left(\begin{array}{ll} 1 & 1\\ i & -i \end{array}\right)$ denotes the transformation matrix connecting linear polarization (LP) bases and CP bases, and $R\left(r\right)=\left(\begin{array}{ll} \cos\left[\alpha \left(r\right)\right] & \sin\left[\alpha \left(r\right)\right]\\ -\sin\left[\alpha \left(r\right)\right] & \cos\left[\alpha \left(r\right)\right] \end{array}\right)$ is the rotating matrix for the meta-atom located at position **r**.

We find that such a meta-device can generate gate-controlled THz wavefronts, as Eq. (1) shows that its Jones-matrix distribution $\tilde{J}\left(r;V_{\mathrm{ext}}\right)$ varies as a function of $V_{\mathrm{ext}}$ . Assume that the incident beam is circularly-polarized with E-field distribution $E^{\text{in}}\left(r\right)=E^{\text{in}}\left(r\right){\hat{e}}_{\sigma }^{\text{in}}$ on the *z* = 0 plane where the meta-device is placed, with ${\hat{e}}_{+}^{\text{in}}=\left(\begin{array}{l} 1\\ 0 \end{array}\right)$ and ${\hat{e}}_{-}^{\text{in}}=\left(\begin{array}{l} 0\\ 1 \end{array}\right)$ denoting incident left circular polarization(LCP) and right circular polarization (RCP), respectively. Then, the reflected wave on the *z* = 0 plane must approximately be $E^{\text{ref}}\left(r;V_{\mathrm{ext}}\right)=\tilde{J}\left(r;V_{\mathrm{ext}}\right)\cdot E^{\text{in}}\left(r\right)$ , which already shows that the reflected wavefront can be controlled by $V_{\mathrm{ext}}$ . Taking the LCP incidence as a specific example, straightforward calculations show that the E-field distribution of the reflected wave right on the *z* = 0 plane is

(2) $$E^{\text{ref}}\left(r;V_{\mathrm{ext}}\right)=A_{a}\left(V_{\mathrm{ext}}\right)e^{i\Phi^{PB}\left(r\right)}E^{\text{in}}\left(r\right){\hat{e}}_{+}^{ref}+A_{n}\left(V_{\mathrm{ext}}\right)E^{\text{in}}\left(r\right){\hat{e}}_{-}^{ref}\text{,}$$

where ${\hat{e}}_{+}^{ref}=\left(\begin{array}{l} 0\\ 1 \end{array}\right)$ and ${\hat{e}}_{-}^{ref}=\left(\begin{array}{l} 1\\ 0 \end{array}\right)$ denote LCP and RCP for the reflected mode, respectively, $\Phi^{PB}\left(r\right)=2\alpha \left(r\right)$ represents the Pancharatnam–Berry (PB) phase [17, 39], and

(3) $$\left\{ \begin{array}{l} A_{a}\left(V_{\mathrm{ext}}\right)=\left[r_{uu}\left(V_{\mathrm{ext}}\right)-r_{vv}\left(V_{\mathrm{ext}}\right)\right]/2\\ A_{n}\left(V_{\mathrm{ext}}\right)=\left[r_{uu}\left(V_{\mathrm{ext}}\right)+r_{vv}\left(V_{\mathrm{ext}}\right)\right]/2 \end{array}\right.\text{,}$$

are the expansion coefficients of the spin-converted abnormal mode modulated by the PB phase $\Phi^{PB}\left(r\right)$ (where subscript a/n denotes the abnormal/normal mode) and the spin-maintained normal mode without the modulations of $\Phi^{PB}\left(r\right)$ , respectively. Most interestingly, while the field patterns of two modes do not change, the coefficients of these two beams (i.e., $A_{a}$ and $A_{n}$ ), however, both sensitively depend on the external gating voltage $V_{\mathrm{ext}}$ . We note that Eq. (2) only represents the electric field distribution of the reflected beam right on the *z* = 0 plane. At a wavefront plane not too far away from the meta-device (i.e., in the near-field (NF) region), we expect that the electric field distribution does not deviate too much from Eq. (2). In the FF region, however, we need to use a Fourier transformation technique [40] to obtain the final FF scattering patterns [see detail derivations in Supplementary Material Sec. I].

Equations (2) and (3) clearly show that the wavefront of reflected beam can be dynamically modulated by varying the gating voltage $V_{\mathrm{ext}}$ . We discuss the following two different scenarios. In the first case where the normal and abnormal modes are spatially well separated in the FF region, gating the graphene can change the intensities ( ${\left|A_{a}\right|}^{2}$ and ${\left|A_{n}\right|}^{2}$ ) of two modes exhibiting distinct wavefronts, thus modulating the reflection property of the whole device dramatically. In the second case where two beams spatially overlap with each other even in the FF region, interference between two beams with $V_{\mathrm{ext}}$ -dependent expansion coefficients must generate a new reflection wavefront (a mixed mode, see Figure 1) which can be continuously modulated by varying the gating voltage.

In the following sections, after designing a meta-atom and experimentally/numerically characterizing how its reflection properties (e.g., $r_{uu}$ and $r_{vv}$ ) vary against the gating voltage on graphene, we then experimentally and numerically demonstrate two graphene meta-devices, precisely corresponding to the two cases discussed above.

## 3 Gating-voltage-dependent properties of the meta-atom

**Figure 2:**
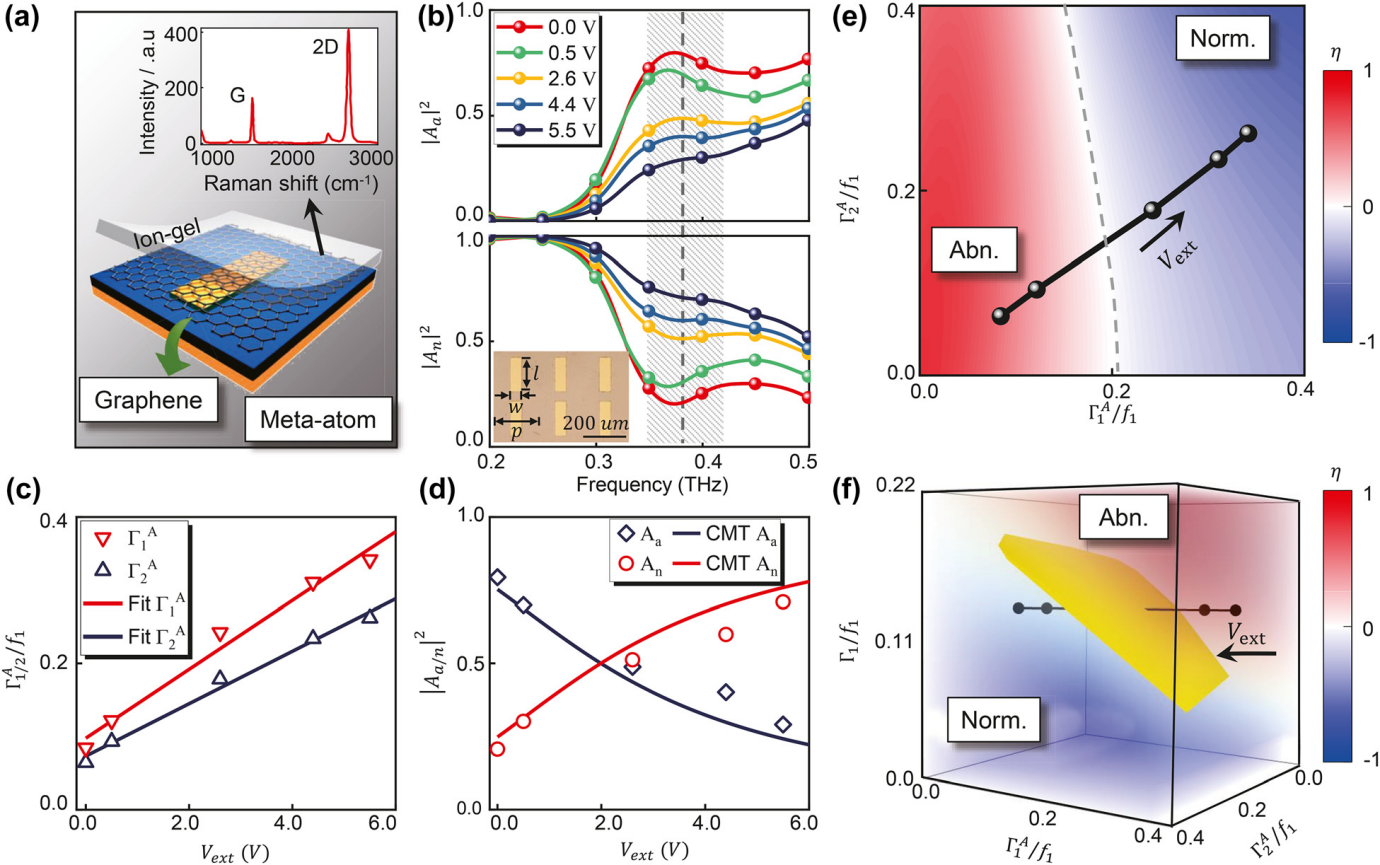
Design and experimental characterizations of graphene gated meta-atoms. (a) A schematic view of an active graphene meta-atom composed of a single layer of graphene deposited on the metallic patch with Raman spectrum of the CVD-grown monolayer graphene as the inset. (b) Measured intensities of abnormal mode ${\left|A_{a}\right|}^{2}$ and normal mode ${\left|A_{n}\right|}^{2}$ vary as a function of frequency under different gate voltages with optical picture of samples in the inset. Here, l=155 μm , w=50 μm , and p=200 μm . (c) Measured and retrieved $\Gamma_{1,2}^{A}∼V_{\mathrm{ext}}$ relationship, and (d) measured and retrieved intensities ${\left|A_{a}\right|}^{2}$ and ${\left|A_{n}\right|}^{2}$ vary as a function of gate voltage with the working frequency fixed as 0.38 THz. (e) The phase diagram of η changes as a function of $\Gamma_{1}^{A}$ and $\Gamma_{2}^{A}$ when $\Gamma_{1}=0.12f_{1}$ . (f) 3D phase diagram of η varies as a function of $\Gamma_{1}^{A}$ , $\Gamma_{2}^{A}$ and $\Gamma_{1}$ , where black circle symbols connected by solid line in (e) and (f) denote different gate voltages, driving the meta-atoms from abnormal-mode-dominant to normal-mode-dominant.

We now design and characterize the optical properties of an active meta-atom that can be used to construct dynamically tunable meta-devices in THz regime. The meta-atom we design is a metal/insulator/metal (MIM) structure with top structure being a rectangle metallic patch, as schematically depicted in Figure 2(a) [41]. The metallic patch is sized 155 × 50 µm, and the lattice constant of the array is chosen as 200 µm. In our fabrication, an Au film is firstly evaporated onto a 47 µm-thick SiO_2_ substrate, and this Au film serves as a perfect reflector for incident THz waves. We then fabricate an array of Au rectangle patches on the layer by using standard optical lithography [see Supplementary Material Sec. II]. We then grow the monolayer graphene on copper foils (Alfa Aesar) by using the standard CVD process. Finally, the graphene sample is transferred onto the structure by wet transfer method and is subsequently covered by a layer of ion-gel liquid. Here, the graphene sample remained uniform after being transferred, and the Raman spectroscopy of a typical graphene sample on a SiO_2_/Si substrate is shown in the inset of Figure 2(a), where Raman spectroscopy measurement also confirmed that the graphene sample was a monolayer. A gate bias $V_{g}$ applied between the graphene layer and a top gate is used to tune the resistance of graphene through varying the carrier density, and in turn, to modulate the loss of the meta-atom. Here, peak resistance of the graphene appears at the voltage $V_{D}$ (i.e., the charge-neutral Dirac point), which is found as $V_{D}\approx 3.7\text{ V}$ for the graphene transferred onto the metasurface [see Supplementary Material Sec. III].

We now characterize the optical responses of the fabricated metasurface under different gate voltages. In our experiments, shining the sample by normally incident THz beams with linear polarizations along two principle axes, we measure the spectra of two reflection coefficients $r_{uu}$ and $r_{vv}$ , and then obtain the strengths of two modes, $A_{a}$ and $A_{n}$ [see Supplementary Material Sec. III]. We note that $V_{\mathrm{ext}}=V_{D}-V_{g}$ determines the doping level of our graphene, and therefore we plot in Figure 2(b) how the spectra of ${\left|A_{a}\right|}^{2}$ and ${\left|A_{n}\right|}^{2}$ vary against $V_{\mathrm{ext}}$ . We find that varying $V_{\mathrm{ext}}$ can generally enlarge ${\left|A_{n}\right|}^{2}$ but suppress ${\left|A_{a}\right|}^{2}$ simultaneously. Set the working frequency as 0.38 THz, we note that whereas ${\left|A_{a}\right|}^{2}$ is much larger than ${\left|A_{n}\right|}^{2}$ at $V_{\mathrm{ext}}=0\text{ V}$ , the trend is gradually reversed as $V_{\mathrm{ext}}$ increases. Such a cross-over can be more clearly seen in Figure 2(d), where ${\left|A_{a}\right|}^{2}$ and ${\left|A_{n}\right|}^{2}$ are depicted as functions of $V_{\mathrm{ext}}$ and we find ${\left|A_{a}\right|}^{2}\approx {\left|A_{n}\right|}^{2}$ at $V_{\mathrm{ext}}=2.6\text{ V}$ . Experimental results are in excellent agreements with FDTD simulated ones [see Supplementary Material Sec. IV]. In our numerical simulations, the optical conductivity of graphene was calculated by the Kubo formula as a function of the Fermi energy $E_{F}$ , which is determined by the applied gate voltage $V_{\mathrm{ext}}$ via $\left|E_{F}\right|=\hbar v_{f}\sqrt{\pi N}$ with $N=\sqrt{n_{0}^{2}+a^{2}V_{\mathrm{ext}}^{2}}$ being the total carrier density and $v_{f}$ the Fermi velocity. Here, $n_{0}$ denotes the residue carrier density and $a\approx 9.02\times 10^{15}\;{\text{m}}^{-2}\;{\text{V}}^{-1}$ is the gate capacitance. In our simulations, we assumed that $n_{0}=1.84\times 10^{15}\;{\text{m}}^{-2}$ and $\tau^{-1}=4\text{ THz}$ with τ denoting the scattering rate.

To understand the physics underlying our experimental results, we use a CMT model to analyze the EM properties of such a tunable meta-atom [42]. The Au film of the structure kills all transmissions through the system, so that we only need to care about the reflection port. Therefore, we can describe the structure by a two-mode two-port CMT model [see Supplementary Material Sec. V]. Through standard CMT analyses, we find the reflection coefficients for two orthogonal directions are given by

(4) $$\left\{ \begin{array}{l} r_{uu}\left(V_{\mathrm{ext}}\right)=C_{1}\left(-1+\frac{2\Gamma_{1}}{i\left(f_{1}-f\right)+\Gamma_{1}+\Gamma_{1}^{A}\left(V_{\mathrm{ext}}\right)}\right)\\ r_{vv}\left(V_{\mathrm{ext}}\right)=C_{2}\left(-1+\frac{2\Gamma_{2}}{i\left(f_{2}-f\right)+\Gamma_{2}+\Gamma_{2}^{A}\left(V_{ext}\right)}\right) \end{array}\right.\text{,}$$

where $C_{1}\left(C_{2}\right)$ denotes the background EM response, $f_{1}\left(f_{2}\right)$ is the resonance frequency, $\Gamma_{1}\left(\Gamma_{2}\right)$ and $\Gamma_{1}^{A}\left(\Gamma_{2}^{A}\right)$ are the decay rates due to radiation and absorption, respectively, for **E** field polarized along $\hat{u}\left(\hat{v}\right)$ axis. We note that different CMT parameters are dictated by different properties of the resonator. Whereas $f_{1}\left(f_{2}\right)$ and $\Gamma_{1}\left(\Gamma_{2}\right)$ are mainly determined by the geometric parameters of the resonator, $\Gamma_{1}^{A}\left(\Gamma_{2}^{A}\right)$ , however, is dictated by the ohmic losses of the whole structure and is certainly closely related to the gate voltage applied on the graphene layer. Therefore, varying $V_{\mathrm{ext}}$ can dramatically modify the two reflection coefficients defined in Eq. (4) through changing the two absorption loss parameters $\Gamma_{1}^{A}$ and $\Gamma_{2}^{A}$ .

All experimental and numerical results [Figure 2(b)] can be well described by the CMT model [Eq. (4)] through carefully determining six fitting parameters $\left(f_{1},f_{2},\Gamma_{1},\Gamma_{2},\Gamma_{1}^{A},\Gamma_{2}^{A}\right)$ . As the starting point, for the Dirac point $\left(V_{\mathrm{ext}}=0\text{ V}\right)$ , we retrieved the fitting parameters as $f_{1}=0.34$ , $f_{2}=0.56$ , $\Gamma_{1}=0.04$ , $\Gamma_{2}=0.14$ , $\Gamma_{1}^{A}=0.03$ , $\Gamma_{2}^{A}=0.02$ , all in unit of THz. The reflection spectra calculated by Eq. (4) with the above fitting parameters are plotted as Figures S19 and S20 in Supplementary Material Sec. V, which agree well with both simulated and experimental results. We note that $\Gamma_{1}^{A}$ and $\Gamma_{2}^{A}$ are non-zero even at the Dirac point $\left(V_{\mathrm{ext}}=0\text{ V}\right)$ , since ohmic losses can be contributed by the Au metal, ion-gel, and the spacer. Using the CMT formulas to fit the reflection spectra obtained at different gate voltages $V_{\mathrm{ext}}$ , we find that $\Gamma_{1}^{A}$ and $\Gamma_{2}^{A}$ are the only parameters tuned by $V_{\mathrm{ext}}$ while all other CMT parameters remain unchanged. This is quite reasonable since the graphene under gating just acts as a tunable loss, as discovered in [41]. We retrieved the $\Gamma_{1/2}^{A}∼V_{\mathrm{ext}}$ relation [see Figure 2(c)] by fitting the CMT spectra with the FDTD ones [see Supplementary Material Sec. V]. Obviously, $\Gamma_{1/2}^{A}$ is an increasing function of $V_{\mathrm{ext}}$ , since increasing $V_{\mathrm{ext}}$ can decrease the resistance of graphene and thus enhance the absorptive decay rate of the resonator. Substituting the retrieved $\Gamma_{1/2}^{A}∼V_{\mathrm{ext}}$ into Eqs. (3) and (4), we obtain the CMT-computed ${\left|A_{a/n}\right|}^{2}∼V_{\mathrm{ext}}$ curves, which are found in very good agreement with experimental and numerical results [see Figure 2(d)].

Defining $\eta =\left[{\left|A_{a}\right|}^{2}-{\left|A_{n}\right|}^{2}\right]/\left[{\left|A_{a}\right|}^{2}+{\left|A_{n}\right|}^{2}\right]$ to quantitatively characterize the reflection property of a meta-atom, we draw the phase diagram in Figure 2(e) depicting how the value of η varies on the $\Gamma_{1}^{A}∼\Gamma_{2}^{A}$ plane, with the radiation loss set as $\Gamma_{1}=0.12f_{1}$ . On the phase plane, we can always find a boundary line with η=0 [dashed line in Figure 2(e)] to divide the whole phase space into two regions with distinct EM response, i.e., the abnormal-mode-dominated region (with η>0 ) and the normal-mode-dominated region (with η<0 ). Interestingly, these boundary lines form a curved surface as radiative loss changes [see Figure 2(f)]. We next depict in the two phase diagrams the trajectories of η as varying $V_{\mathrm{ext}}$ [see symbols in Figure 2(e) and (f)]. Clearly, increasing $V_{\mathrm{ext}}$ can always drive the meta-atom to move from the abnormal-mode-dominated to the normal-mode-dominated one, crossing the phase boundary lines [see Figure 2(e) and (f)].

The underlying physics is ultimately connected to the competition between the absorption loss and the radiation loss. According to the CMT model in Eq. (4), we find that the resonance mode for the $E∥\hat{u}$ polarization is at $f_{1}$ = 0.34 THz, while the resonance mode for another polarization is at $f_{2}$ = 0.57 THz. Therefore, at the vicinity of the working frequency 0.38 THz which is very close to $f_{1}$ , whereas the reflection for the $E∥\hat{u}$ polarization exhibits a zero phase at $V_{\mathrm{ext}}$ = 0 V dictated by the magnetic resonance, the reflection phase for the $E∥\hat{v}$ polarization is nearly π since the relevant resonance is far way [see simulated field distribution in Figure S13]. As $V_{\mathrm{ext}}$ increases, the $E∥\hat{u}$ resonance mode undergoes a transition from an underdamped resonator to an overdamped one [41], making the reflection phase gradually changing from 0 to π . Meanwhile, the reflection phase for another polarization is essentially un-affected by the doping, since the working frequency is far away from the resonance for this polarization [see Figure S13]. Simply speaking, gating the graphene can dramatically change the reflection property (particularly the phase) of one polarization but has little effect on the reflection property of another polarization, which finally leads to the enhancement of ${\left|A_{n}\right|}^{2}$ and the diminishment of ${\left|A_{a}\right|}^{2}$ , and in turn, the dramatic modulations on the functionality of the meta-atom. As a short summary to this section, the CMT model reveals the critical role of graphene serving as the tunable loss to drive the meta-atom transit from two completely distinct EM responses.

## 4 Graphene meta-deflector with switchable beam directions

**Figure 3:**
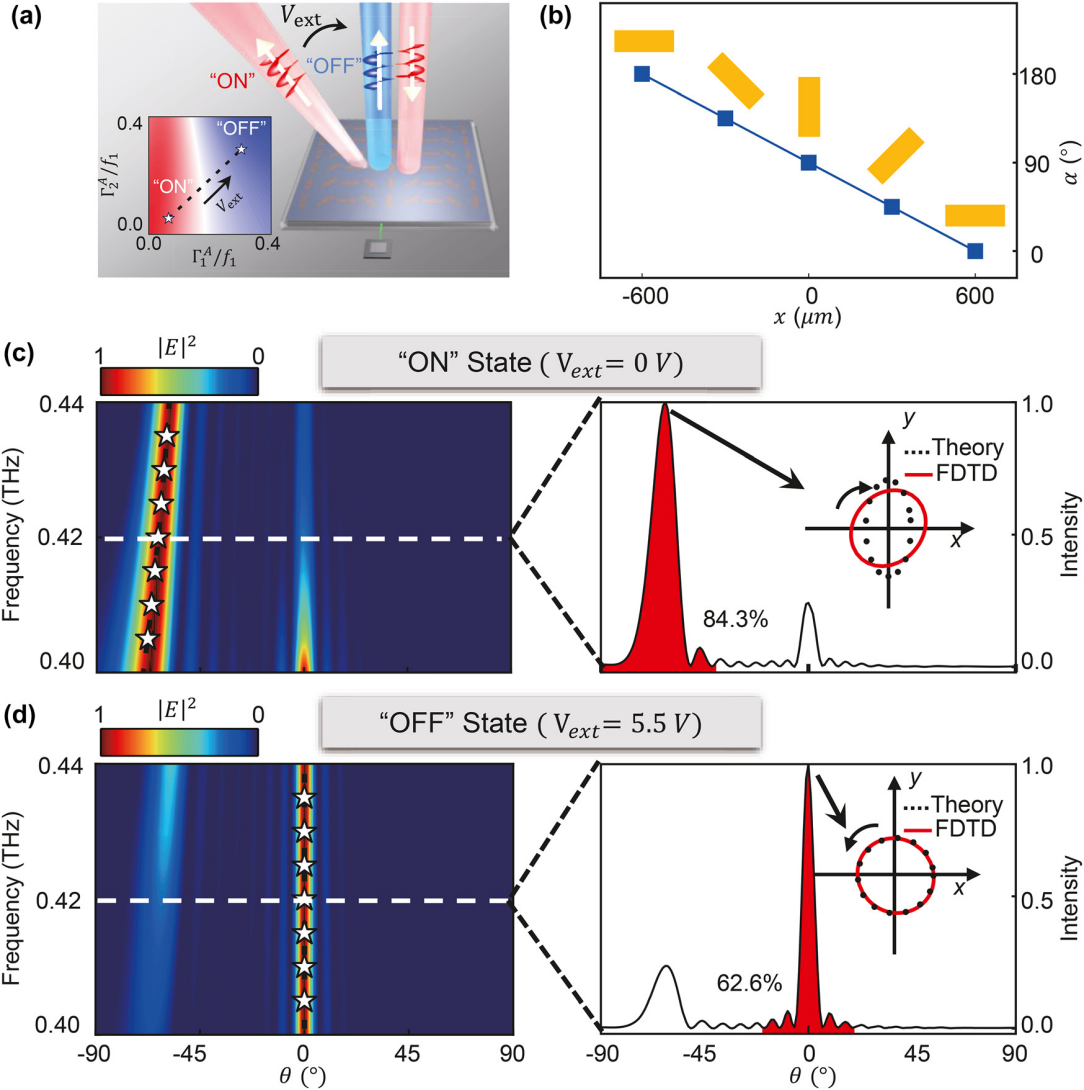
Simulated results of the gate-controlled beam deflector with two pre-designed switchable beam directions. (a) Schematic of gate-tuned beam deflector constructed by identical meta-atoms with a linear gradient orientation angle profile. The inset illustrates the phase diagram of η changes as a function of $\Gamma_{1}^{A}$ and $\Gamma_{2}^{A}$ , where the dotted line denotes the evolution of η varying with gate voltage $V_{\mathrm{ext}}$ . (b) The orientation angles of meta-atoms along the *x*-direction. Simulated angular distribution of FF scattered electric field intensities for reflected waves under two different gate voltages (c) $V_{\mathrm{ext}}$ = 0 V (“ON” state) and (d) $V_{\mathrm{ext}}=V_{\max}$ (“OFF” state), with the corresponding deflection efficiencies and the scattered polarizations at the working frequency of 0.42 THz shown in the right-hand panels.

The first meta-device that we realize is a gate-controlled beam deflector which can switch the reflected THz beam between two pre-designed directions, as shown in Figure 3(a). To achieve this goal, we construct our meta-device with a series of designed meta-atoms exhibiting orientation angles described by α(r)=ξx/2 where $\xi =-0.89k_{0}$ denotes the linear phase gradient [see Figure 3(b)]. We follow the theory described in Section 2 and use a Fourier-transformation technique to calculate the FF scattered electric field as the metasurface is shined by a normally incident LCP plane wave. The calculated E-field can be approximately written as

(5) $$\begin{array}{l} E^{ref}\left(k;V_{\mathrm{ext}}\right)=A_{a}\left(V_{\mathrm{ext}}\right)\left[\delta \left(k_{x}-\xi \right)\delta \left(k_{y}\right)\right]\psi_{+}^{ref}\left(k\right)\\ \;+A_{n}\left(V_{\mathrm{ext}}\right)\left[\delta \left(k_{x}\right)\delta \left(k_{y}\right)\right]\psi_{-}^{ref}\left(k\right)\text{,} \end{array}$$

where $\psi_{\sigma }^{ref}\left(k\right)$ denote the eigen wave functions of the scattered LCP and RCP waves [see concrete form of $\psi_{\sigma }^{ref}\left(k\right)$ in Supplementary Material Sec. I]. Equation (5) reveals that the reflected abnormal mode can acquire a tangential wave-vector $k_{x}=\xi$ , while normal mode is still a specular reflection beam, indicating that these two modes can be redirected to two different directions

(6) $$\left\{ \begin{array}{l} \theta_{a}=\sin^{-1}\left[\xi /k_{0}\right]\\ \theta_{n}=0 \end{array}\right.\text{,}$$

where $\theta_{a}\left(\theta_{n}\right)$ denotes the deflection angle for abnormal (normal) modes. In fact, according to our rigorous theory described in Supplementary Material Sec. I, the reflected polarization in FF region for non-zero bending angle is slightly tilted from the origin one at *z* = 0 plane, which comes from the projection of the normal and abnormal modes between NF and FF regions.

Equations (5) and (6) predict that we can dynamically modulate the reflection beam by gating the graphene. At the Dirac point $\left(V_{\mathrm{ext}}=0\text{ V}\right)$ , we have ${\left|A_{a}\left(0\right)\right|}^{2}\gg {\left|A_{n}\left(0\right)\right|}^{2}$ , and thus the reflected beam is along the anomalous-deflection angle $\left(\theta_{a}\right)$ exhibiting left circular polarization. We define this state as the “ON” state. Meanwhile, under the highest gate voltage $\left(V_{\mathrm{ext}}=V_{\max}\right)$ , we have ${\left|A_{a}\left(V_{\max}\right)\right|}^{2}\ll {\left|A_{n}\left(V_{\max}\right)\right|}^{2}$ and thus the reflected beam mainly contains normal mode propagating along the spectacular reflection direction $\left(\theta_{n}\right)$ exhibiting right circular polarization. We denote this state as the “OFF” state for the meta-device. Therefore, varying the gate voltage can help us efficiently switch the functionality of the meta-device designed [see the inset of Figure 3(a)].

We perform full-wave simulations on the designed meta-device to verify the above predictions. Figure 3(c) and (d) illustrate the simulated scattering patterns of the meta-device under the illumination of a normally incident LCP wave, as we vary the gate voltage from 0 to 5.5 V. In the case of $V_{\mathrm{ext}}$ = 0 V, simulations show that the reflected beam is mainly along the abnormal deflection-angle $\theta_{a}=-63{}^{\circ}$ with polarization being LCP approximately, while the specular reflection is very weak [see Figure 3(c)]. Meanwhile, in the case of $V_{\mathrm{ext}}$ = 5.5 V, we find that the reflection mainly consists of the component with polarization being RCP, as predicted [see Figure 3(d)]. Moreover, to quantitatively evaluate the efficiency of our meta-device, we integrate the abnormal (normal) reflected beam power flow (normalized by the total output power) and define the obtained value as the efficiency of “ON” (“OFF”) state for the proposed meta-deflector. At the working frequency (0.42 THz), The meta-device achieves an efficiency of 84.3% for the “ON” state and 62.6% for the “OFF” state, which are in nice agreement with theoretical calculations [see Supplementary Material Sec. I].

**Figure 4:**
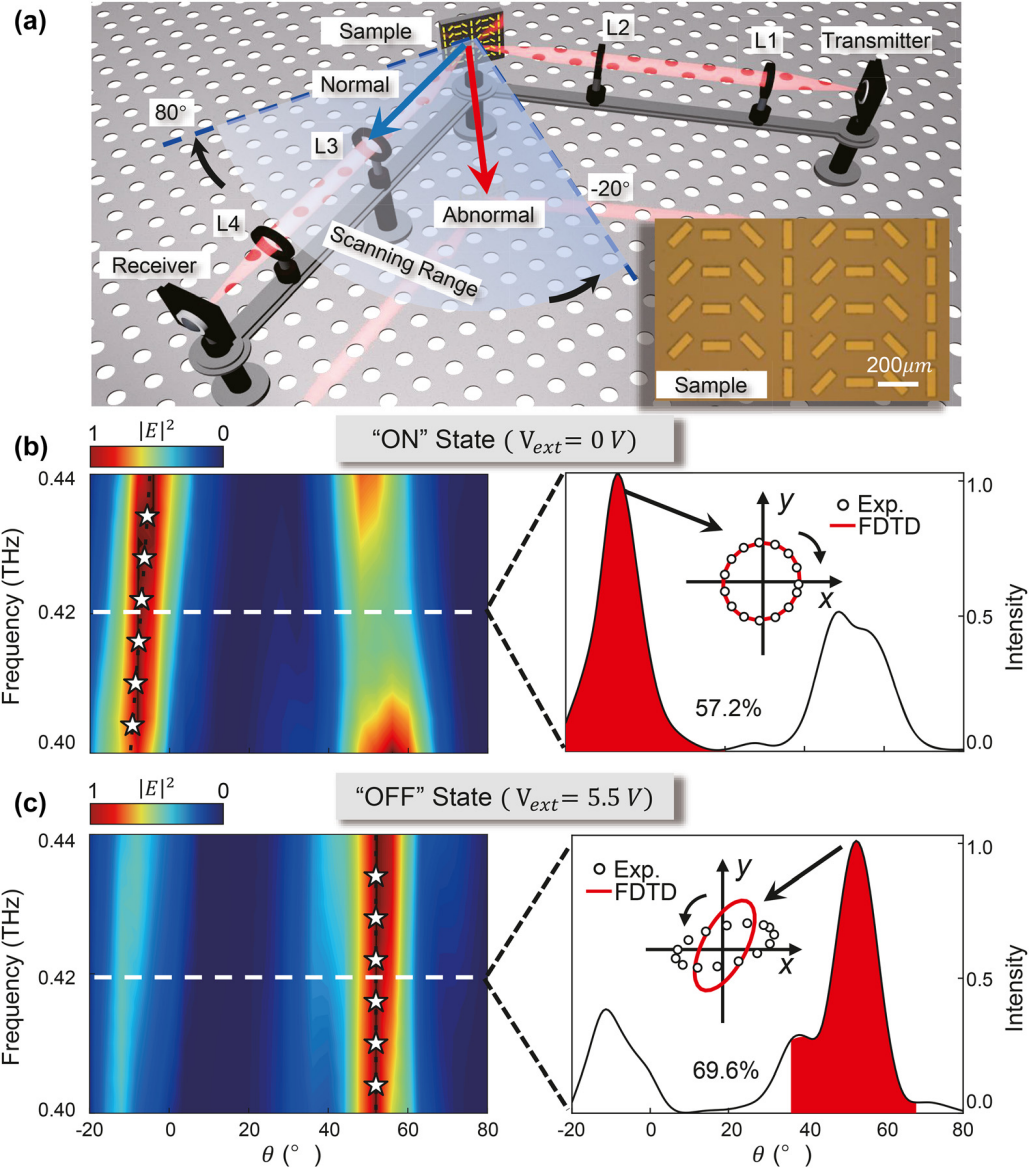
Experimental results of the gate-controlled beam deflector with two pre-designed switchable beam directions. (a) Schematic of the experimental setup to characterize the tunable beam deflector. Inset presents the optical photography for the fabricated sample. The left-hand panels in (b and c) show the measured angular distribution of FF scattered electric field intensities of reflected waves for (b) “ON” state with $V_{\mathrm{ext}}$ = 0 V and (c) “OFF” state with $V_{\mathrm{ext}}=V_{\max}$ , with the corresponding deflection efficiencies and the scattered polarizations at 0.42 THz shown in the right-hand panels.

We fabricate out the graphene meta-device following the same process as described in Section 2, and the characterize its $V_{\mathrm{ext}}$ -dependent reflection properties by an angle-resolved THz time-domain spectroscopy (THz-TDS) system [see Figure 4(a)]. Since our TDS system does not allow us to measure the normally reflected THz signals, we choose to shine the meta-device at the incident angle of 50°. Under such a condition, deflection angles for normal and abnormal modes are calculated as 50° and −7.3°, respectively, according to the generalized Snell’s law. On the other hand, we also characterize the polarization properties of the reflected beams, by simply rotating the linearly polarized terahertz transmitter and receiver [see Supplementary Material Sec. III]. Left panels in Figure 4(b) and (c) depict the measured scattering patterns of the meta-device with graphene gated at $V_{\mathrm{ext}}$ = 0 V and $V_{\mathrm{ext}}$ = 5.5 V, respectively, under the LCP incidence at 50°. Notably, the beam reflected by our meta-device changes from the abnormal-mode-dominated (along −7.3° direction) to normal-mode-dominated (along 50° direction), as voltage changes from $V_{\mathrm{ext}}$ = 0 V to $V_{\mathrm{ext}}$ = 5.5 V. Such a conclusion is more clearly seen in the right panels of Figure 4(b) and (c), where the measured normalized scattering patterns of the meta-device under two different gate voltages are depicted, at the working frequency of 0.42 THz. Quantitative evaluations reveal that our meta-device achieves an efficiency of 57.2% for the “ON” state and an efficiency of 69.6% for the “OFF” state. We find the working frequency of our device is slightly shifted from 0.38 THz (for the meta-atom exhibiting the best performance) to 0.42 THz. Such shift might be due to errors in sample fabrication and differences in ion-gel gating in two different experiments [see geometrical parameters for meta-atoms in Supplementary Material Sec. IV]. Moreover, we also experimentally characterize the polarization states of the deflected beams [see Supplementary Material Sec. III]. Measured polarization patterns of the reflected THz beams in “ON” and “OFF” states are shown as open circles in right panels of Figure 4(b) and (c), which are in reasonable agreement with theoretical and numerical results.

## 5 Graphene-tuned vectoral beam generator with variable polarizations

**Figure 5:**
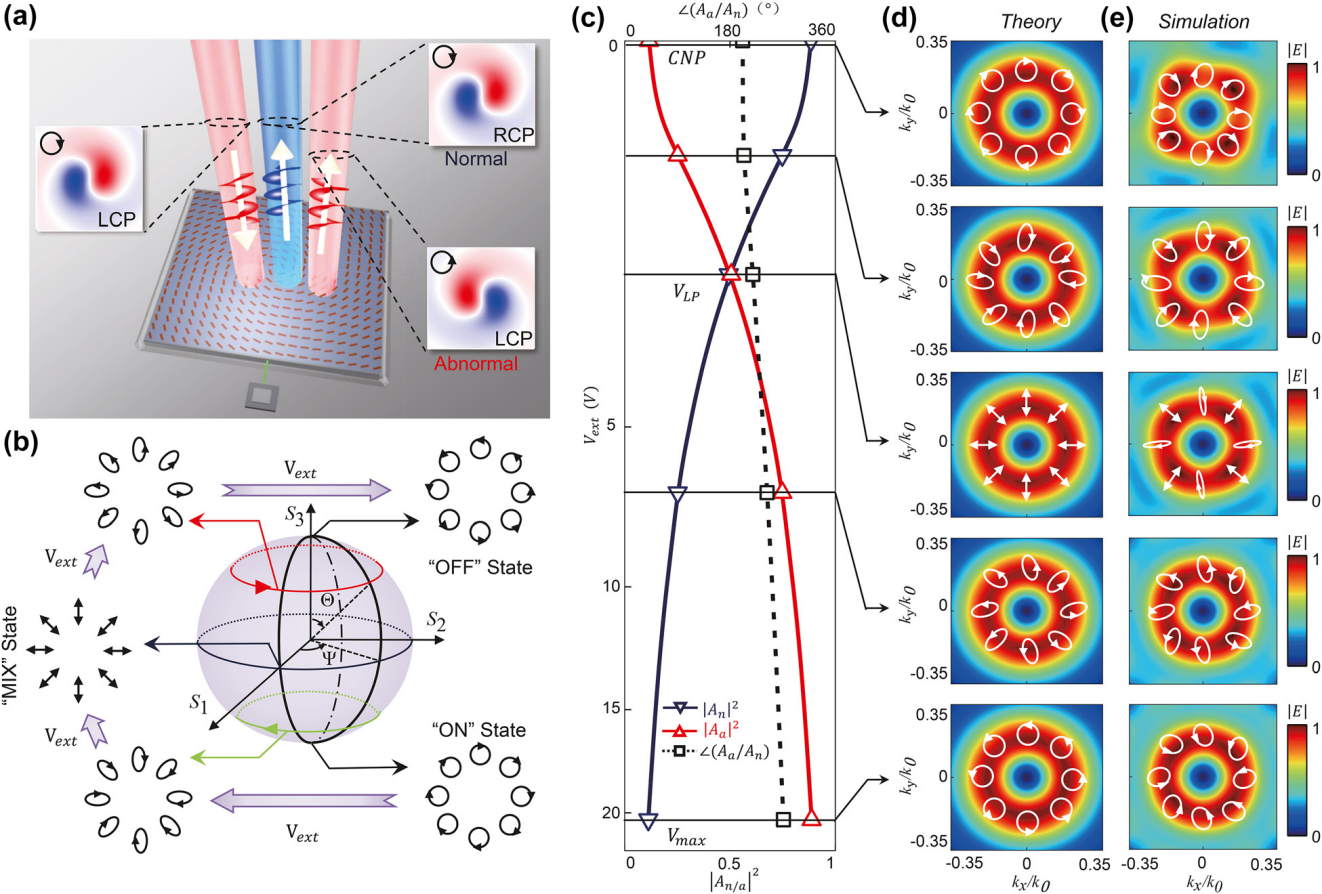
Simulated results of the graphene-controlled meta-device which can generate vectorial THz beams with continuously varying polarization distributions upon gating. (a) Schematic for gate-tuned vectorial beam generator. Such meta-device is illuminated by a normally incident LCP Laguerre–Gaussian beam with topological charge, and the reflected beams are two beams of Laguerre–Gaussian with topological charge l=−1 for normal mode (“ON” state) and l=1 for abnormal mode (“OFF” state) with two opposite circular polarizations. (b) The evolution of the polarizations for the interfered beam (“MIX” state) on Poincare’s sphere as gate voltage changes. (c) As the intensities and phase difference of two modes gate voltage evolving over gate voltage, the corresponding (d) theoretical calculated and (e) simulated scattering electric filed distributions $\left|E^{ref}\left(k;V_{\mathrm{ext}}\right)\right|$ and the polarization distributions.

We proceed to demonstrate a device of the second type as described in Section 2. Specifically, upon excitation of a normally incident Laguerre–Gaussian (LG) beam with a circular polarization, the beam reflected by the proposed meta-device is a *vectorial* beam polarization distribution continuously modulated by $V_{\mathrm{ext}}$ [see Figure 5(a)]. To achieve this end, in constructing the meta-device, we arrange the meta-atoms with orientation angles satisfying $\alpha \left(r\right)=\phi +\alpha_{0}$ with $\phi =\tan^{-1}\left(y/x\right)$ being the azimuthal angle and $\alpha_{0}$ a constant. Suppose that the excitation beam is a normally incident LCP LG-beam carrying a topological charge of l=−1 , $E^{in}\left(r\right)=\exp\left[-\left(x^{2}+y^{2}\right)/w_{0}^{2}\right]\exp\left[-i\phi \right]$ with $w_{0}$ denoting the beam waist, the beam reflected by our meta-device must exhibit an E-field distribution

(7) $$\begin{array}{l} E^{ref}\left(r;V_{\mathrm{ext}}\right)=A_{a}\left(V_{\mathrm{ext}}\right)e^{i\left[\phi +2\alpha_{0}\right]}e^{-\left(x^{2}+y^{2}\right)/w_{0}^{2}}{\hat{e}}_{+}^{ref}\\ \;+A_{n}\left(V_{\mathrm{ext}}\right)e^{-i\phi }e^{-\left(x^{2}+y^{2}\right)/w_{0}^{2}}{\hat{e}}_{-}^{ref}\text{.} \end{array}$$

on the *z* = 0 plane. Obviously, both normal and abnormal modes propagate along the same direction, since the designed meta-device does not provide a phase gradient along any Cartesian axis. Meanwhile, the meta-device does provide a phase gradient along the azimuthal direction to the abnormal mode, making the reflected abnormal mode possessing a topological charge of l=+1 [see Eq. (7)], different from the normal mode exhibiting l=−1 [see inset in Figure 5(a)]. We still define the state that only abnormal mode (with l=1 ) exists as the “ON” state and the state that only normal mode (with l=−1 ) exists as the “OFF” state. Different from the first device demonstrated in Section 3, here in this case the two states can interfere with each other in the FF, thus generating a “MIX” state depending sensitively on the amplitudes and phases of two coefficients $A_{a}$ and $A_{n}$ . Moreover, since $A_{a}$ and $A_{n}$ are efficiently tuned by $V_{\mathrm{ext}}$ , we thus expect that the final wavefront of the reflected beam can be continuously tuned.

Quantitatively, the “MIX” state can be expressed as

(8) $$E^{ref}\left(r;V_{\mathrm{ext}}\right)=e^{-\left(x^{2}+y^{2}\right)/w_{0}^{2}}A\left(V_{\mathrm{ext}}\right)\left(\begin{array}{l} e^{-i\Psi \left(r;V_{\mathrm{ext}}\right)/2}\;\cos\left(\Theta \left(V_{\mathrm{ext}}\right)/2\right)\\ e^{i\Psi \left(r;V_{\mathrm{ext}}\right)/2}\;\sin\left(\Theta \left(V_{\mathrm{ext}}\right)/2\right) \end{array}\right)\text{,}$$

where $A\left(V_{\mathrm{ext}}\right)=\sqrt{{\left|A_{a}\left(V_{\mathrm{ext}}\right)\right|}^{2}+{\left|A_{n}\left(V_{\mathrm{ext}}\right)\right|}^{2}}e^{i\left[\arg\left[A_{a}\left(V_{\mathrm{ext}}\right)\right]+\arg\left[A_{n}\left(V_{\mathrm{ext}}\right)\right]+2\alpha_{0}\right]/2}$ represents the coefficients of the overlapped reflected beam, and (Θ,Ψ) determine the polarization state on Poincare’s sphere with $\Theta \left(V_{\mathrm{ext}}\right)=2\;\cos^{-1}\left[\left|A_{n}\left(V_{\mathrm{ext}}\right)\right|/\left|A_{a}\left(V_{\mathrm{ext}}\right)\right|\right]$ and $\Psi \left(r;V_{\mathrm{ext}}\right)/2=\alpha \left(r\right)+\left[\arg\left[A_{a}\left(V_{\mathrm{ext}}\right)\right]-\arg\left[A_{n}\left(V_{\mathrm{ext}}\right)\right]\right]/2$ . Interestingly, we find the polar angle Ψ/2 of the polarization state is a function of r , indicating that the reflected beam must be a vectorial one with an *inhomogeneous* polarization distribution.

We now analyze Eq. (8) to see how the reflected wavefront changes as a function of gate voltage. For the meta-atom studied in Section 2, increasing $V_{\mathrm{ext}}$ can lead to enhancement of $\left|A_{n}\right|$ and diminishment of $\left|A_{a}\right|$ simultaneously. Therefore, as $V_{\mathrm{ext}}$ increases, we expect that Θ must move from the south pole to the north pole on Poincare’s sphere [see Figure 5(b)], and reaches the equatorial plane at $V_{\mathrm{ext}}=V_{\mathrm{LP}}$ yielding ${\left|A_{n}\left(V_{\mathrm{LP}}\right)\right|}^{2}={\left|A_{a}\left(V_{\mathrm{LP}}\right)\right|}^{2}$ [see Figure 5(c)]. Meanwhile, the distribution of polarization angle $\left\{\Psi \left(r;V_{\mathrm{ext}}\right)/2\right\}$ can also be efficiently tuned by $V_{\mathrm{ext}}$ through the term $\left[\arg\left[A_{a}\left(V_{\mathrm{ext}}\right)\right]-\arg\left[A_{n}\left(V_{\mathrm{ext}}\right)\right]\right]$ . Specifically, set the initial angle as $\alpha_{0}=\left[\arg\left[A_{n}\left(V_{\mathrm{LP}}\right)\right]-\arg\left[A_{a}\left(V_{\mathrm{LP}}\right)\right]\right]/2$ , we find that in the case of $V_{\mathrm{ext}}=V_{\mathrm{LP}}$ the local polarization direction is just $\Psi \left(r;V_{\mathrm{LP}}\right)/2=\phi$ , which precisely represents a cylindrically polarized vectorial beam [see Figure 5(d)]. Further varying $V_{\mathrm{ext}}$ , we find that the polarization distribution of the interfered beam can cover an arbitrary weft on Poincare’s sphere, as shown in Figure 5(b).

We finally use the realistic meta-atom studied in Section 2 as a building block to construct this meta-device. Based on the $A_{n}\text{∼}V_{\mathrm{ext}}$ and $A_{a}\text{∼}V_{\mathrm{ext}}$ relations retrieved from the numerical simulation results on the realistic meta-atom, we can determine $V_{\mathrm{LP}}=1.83\;V$ and thus the initial $\alpha_{0}=\left[\arg\left[A_{n}\left(V_{\mathrm{LP}}\right)\right]-\arg\left[A_{a}\left(V_{\mathrm{LP}}\right)\right]\right]/2=-110{}^{\circ}$ . We then perform full-wave simulations to demonstrate the predicated functionality of the device upon gating, again with retrieved $A_{n}\text{∼}V_{\mathrm{ext}}$ and $A_{a}\text{∼}V_{\mathrm{ext}}$ relations put into Eq. (8). Figure 5(d) and (e) plot, respectively, the FF scattered-field distributions $\left|E^{ref}\left(k;V_{\mathrm{ext}}\right)\right|$ and the polarization patterns of the beams reflected by our meta-device at different gate voltages $V_{\mathrm{ext}}$ , obtained by theoretical calculations [Eq. (8)] and full-wave simulations [see Figure 5(c)]. Singular points in the field patterns [Figure 5(d) and (e)] signify the existences of vortex wavefronts, while the simulated local polarization distributions at different $V_{\mathrm{ext}}$ are in good agreement the theoretically calculated results. In particular, full simulations demonstrate that the polarization distribution of the generated THz vectorial beam indeed changes continuously as $V_{\mathrm{ext}}$ varies, based on the global-tuning mechanism proposed in this paper.

## 6 Conclusions and discussions

In summary, we have proposed *globally tuned* graphene meta-devices to achieve dynamical terahertz wavefront control and experimentally demonstrate them in the THz regime. We use the CMT analysis to establish a generic phase diagram for such meta-device, revealing that the gated graphene as tunable loss can drive the whole meta-device to transit between two functional phases exhibiting completely distinct reflected wavefronts. Based on such mechanism, we experimentally demonstrate a tunable meta-device with switchable deflection angle controlled by graphene gate voltage. We finally numerically present a graphene-controlled meta-device which can generate vectorial THz beams with continuously varying polarization distributions upon gating. Our results open the gateway to terahertz tunable meta-devices control global tuning fashion, which may inspire many future works on both fundamental and application sides of research (e.g., THz radar, vectorial beam coded communication, tunable spin-Hall momentum shift, etc.).

We mention several important points before closing this section. Here, we note that the modulation efficiency is hampered by the dynamic range of absorptive loss for our fabricated meta-device (around 60%), which is not an intrinsic limitation of the design strategy. If we adopt other architecture with lower system absorption (e.g., back-gate control) and improved-quality graphene [43], the modulation range can be efficiently improved to 80% [see Supplementary Material Sec. VI]. Moreover, we note that our proposed meta-devices only use the geometric phase profiles in wavefronts control, making the scattering field for the normal mode always being a specular reflection. This is not an intrinsic limitation of our global tuning mechanism. If we further introduce a resonance phase, together with PB phase, into EM responses for each meta-atom, the scattering behaviors of two modes can be completely pre-designable to achieve more flexible wavefront control phenomena [see Supplementary Material Sec. VII]. We expect more plausible applications of our proposed strategy.

## Supplementary Material

Supplementary Material
