# Supplementary material for: Gate-tuned graphene meta-devices for dynamically controlling terahertz wavefronts
Source: Nanophotonics. 2022 Mar 31;11(9):2085–96. doi: 10.1515/nanoph-2021-0801 (PMC11501680; doi:10.1515/nanoph-2021-0801)
Supplement: Supplementary file 1 — Supplementary Material [file j_nanoph-2021-0801_suppl.docx]

Supplementary material for

**Gate-tuned graphene meta-devices for dynamically controlling terahertz wavefronts**

**Qiushi Li^1,†^, Xiaodong Cai^1,†^, Tong Liu^2,†^, Min Jia^2^, Qiong Wu^2^, Haoyang Zhou^2^, Huanhuan Liu^3^, Qianqian Wang^1^, Xiaohui Ling^4^, Cong Chen^5^, Fan Ding^6^, Qiong He^2,7,8^, Yuanbo Zhang^2,7,8^, Shiyi Xiao^1,*^, and Lei Zhou^2,7,8*^**

^1^ Key Laboratory of Specialty Fiber Optics and Optical Access Networks, Joint International Research Laboratory of Specialty Fiber Optics and Advanced Communication, Shanghai Institute for Advanced Communication and Data Science, Shanghai University, Shanghai 200444, China

^2^ State Key Laboratory of Surface Physics, Key Laboratory of Micro and Nano Photonic Structures (Ministry of Education) and Physics Department, Fudan University, Shanghai 200433, China

^3^ Department of Electrical and Electronic Engineering, Southern University of Science and Technology, Shenzhen 518055, China

^4^ College of Physics and Electronic Engineering, Hengyang Normal University, Hengyang 421002, China

^5^ School of Electronic Information, Wuhan University, Wuhan 430072, China

^6^ China Ship Development and Design Center, Wuhan 430064, China

^7^ Fudan University, Academy for Engineering and Technology, Shanghai 200433, China

^8^ Collaborative Innovation Centre of Advanced Microstructures, Nanjing 210093, China

*Corresponding Authors: [phxiao@shu.edu.cn](mailto:phxiao@shu.edu.cn), [phzhou@fudan.edu.cn](mailto:phzhou@fudan.edu.cn)

†These authors contributed equally to this work.

**List of contents**

[**I. Theoretical calculation of FF scattering patterns** 1](#_Toc97722561)

[**II. Sample Fabrication** 4](#_Toc97722562)

[**III. Experimental characterization** 6](#_Toc97722563)

[**IV. Detailed information for full-wave simulations** 9](#_Toc97722564)

[**V. CMT fitting for meta-atoms** 14](#_Toc97722565)

[**VI. Possible methods for further improving the efficiency** 15](#_Toc97722566)

[**VII. Flexible wavefront control, based on resonance and PB phases** 17](#_Toc97722567)

1. **Theoretical calculation of FF scattering patterns**
   1. **Definitions of far-field coordinate, unit vectors and wavevectors**


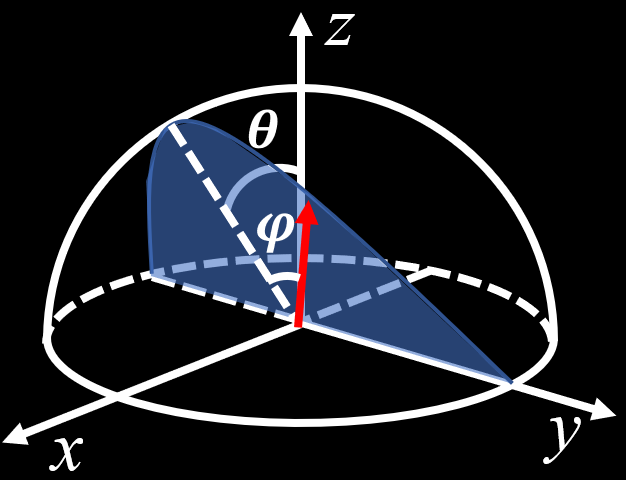


**Figure S1:** Far-field coordinate system

As shown in Figure S1, in such a far-field coordinate system:

, (S1)

with  denoting the reflected elevation angle and azimuthal angle for scattered waves. Consequently, for any scattered waves, the unit vectors for horizontal and vertical polarizations can be expressed as:

, (S2)

and the unit vectors of circular polarizations for any scattered waves are:

,(S3)

where $\hat{e}_{\sigma'}^{ref}=\left(\hat{x}-\sigma'i\hat{y}\right)/\sqrt{2}$ denotes the unit vectors of circular polarizations for normally scattered waves.

In such a coordinate system, the wave vector components for any scattered waves are:

. (S4)

**1.2 Calculation of the FF scattering fields**

As illustrated by Eq. (2) in the main text, we obtain the electric field distributions at *z*=0 plane for LCP incidence as:

$E^{ref}\left(r;V_{ext}\right)=A_{a}\left(V_{ext}\right)e^{i\Phi^{PB}(r)}E^{in}\left(r\right)\hat{e}_{+}^{ref}+A_{n}\left(V_{ext}\right)E^{in}\left(r\right)\hat{e}_{-}^{ref}$, (S5)

where $\hat{e}_{\sigma}^{ref}$ are the unit vectors for normally reflected waves with two opposite polarizations. Considering the Eigen wave function of scattered LCP and RCP waves as:

$\psi_{\sigma}^{ref}\left(k\right)=e^{ik\cdot r}\hat{e}_{\sigma,k}^{ref}$, (S6)

where $\hat{e}_{\sigma,k}^{ref}$ denotes the unit vectors for scattered waves with different wave vectors $k$ for both circular polarizations. Through projecting the aperture field distributions onto the conjugate of $\psi_{\sigma}^{ref}\left(k\right)$ and summing it, we can obtain the expansion coefficients for the two scattered polarization components as:

$\begin{matrix} E^{ref}\left(k;V_{ext}\right)\text{=}\left(\begin{matrix} E_{a}^{ref}\left(k;V_{ext}\right) \\ E_{n}^{ref}\left(k;V_{ext}\right) \end{matrix}\right)=\left(\begin{matrix} \iint\left[\psi_{a}^{ref}\left(k\right)\right]^{*}E^{ref}\left(r;V_{ext}\right)dxdy \\ \iint\left[\psi_{n}^{ref}\left(k\right)\right]^{*}E^{ref}\left(r;V_{ext}\right)dxdy \end{matrix}\right) \\ =\left(\begin{matrix} \left[\hat{e}_{+,k}^{ref}\right]^{*}\hat{e}_{+}^{ref} & \left[\hat{e}_{+,k}^{ref}\right]^{*}\hat{e}_{-}^{ref} \\ \left[\hat{e}_{-,k}^{ref}\right]^{*}\hat{e}_{+}^{ref} & \left[\hat{e}_{-,k}^{ref}\right]^{*}\hat{e}_{-}^{ref} \end{matrix}\right)\left(\begin{matrix} A_{a}\left(V_{ext}\right)\iint e^{-ik\cdot r}\left[e^{i\Phi^{PB}(r)}E^{in}\left(r\right)\right]dxdy \\ A_{n}\left(V_{ext}\right)\iint e^{-ik\cdot r}\left[E^{in}\left(r\right)\right]dxdy \end{matrix}\right) \end{matrix}$, (S7)

where we find that the total scattered field is divided into two elements, the first element corresponds to the projection of polarizations between the normally reflected and arbitrary scattered waves, and the second element corresponds to Fourier transformation. Thus, the concrete form of $P\left(k\right)$ is expressed as:

, (S8)

where . By assuming the polar angle of scattered waves are small enough, the $P\left(k\right)$ can be approximated as a diagonalized matrix:

$P\left(k\right)=\left(\begin{matrix} 1 & 0 \\ 0 & 1 \end{matrix}\right)$. (S9)

Thus, the total FF scattering field can be approximated as:

$\begin{matrix} E^{ref}\left(k;V_{ext}\right)=A_{a}\left(V_{ext}\right)F\left\{E^{in}\left(r\right)e^{i\Phi^{PB}(r)}\right\}\psi_{+}^{ref}\left(k\right) \\ +A_{n}\left(V_{ext}\right)F\left\{E^{in}\left(r\right)\right\}\psi_{-}^{ref}\left(k\right) \end{matrix}$, (S10)

which corresponds to Eq. (5) in the main text.

**1.3 Theoretical calculation for the meta-defector in Sec. IV**

For the beam-deflector in Sec. IV in the main text, the phase gradient is designed as $\xi=-0.89k_{0}$. By illuminating such a meta-device with a normal LCP incident plane wave ($E^{in}\left(r\right)=1$), we can calculate the simplified theoretical form for the total scattering electric field as:

$E^{ref}\left(k;V_{ext}\right)=\left(\begin{matrix} A_{a}\left(V_{ext}\right)\left[\delta\left(k_{x}+0.89k_{0}\right)\delta\left(k_{y}\right)\right] \\ A_{n}\left(V_{ext}\right)\left[\delta\left(k_{x}\right)\delta\left(k_{y}\right)\right] \end{matrix}\right)$, (S11)

which corresponds to Eq. (5) in the main text. Based on Eq. (S7), we calculate the angular distribution of the scattered electric field intensities and the deflected polarizations at $A_{a}\left(0\right)>>A_{n}\left(0\right)$ (“ON” state) and $A_{a}\left(V_{max}\right)<<A_{n}\left(V_{max}\right)$ (“OFF” state), as shown in Figure S2. Here, in order to obtain quantitative estimations of the deflection efficiencies, we integrate the widths of the deflected beams to obtain the total deflection powers, and integral the total power over the entire beam width as the reference signal. The deflection efficiency is defined as the ratio between these two components, which are denoted by the retrieved values shown in Figure S2.


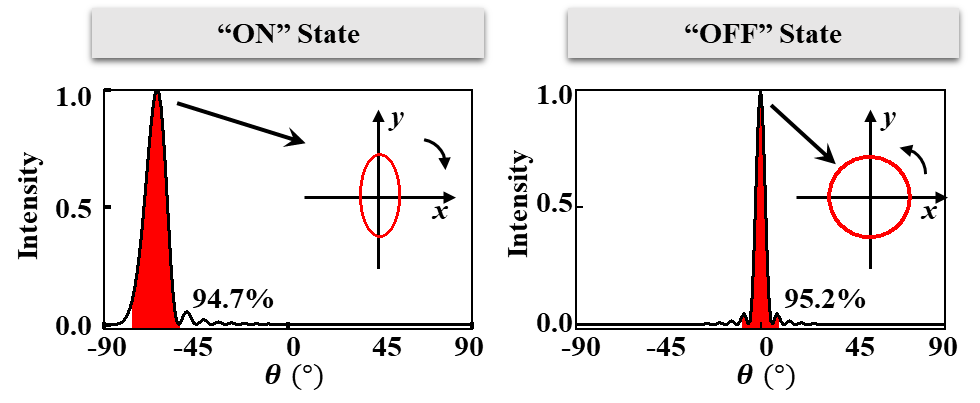


**Figure S2:** Theoretical angular distribution of scattered electric field intensities, and deflection efficiencies and polarizations when the meta-deflector is at “ON” state and “OFF” state.

1. **Sample Fabrication**

The fabrication of graphene metasurface devices included typical microfabrication techniques and a CVD-grown graphene transfer method. All metallic structures (meta-atom layer, back layer) were composed of 60 nm thick gold (Au) with a 10 nm thick chromium (Cr) adhesion layer from conventional electron-beam (E-beam) evaporation. Through the commonly used method of wet transfer, the single-layer graphene grown on the copper foil by the CVD process is directly transferred to the metasurface.

As a gate dielectric, our employed ion-gel is composed of 1-Ethyl-3-methylimidazolium bis (trifluoromethylsulfonyl) imide ([EMIM][TFSI]), and poly (styrene-ethylene oxide-b-styrene (PS-PEO-PS). The formation method is as follows: in a glove box, weigh 42 mg of PS-PEO-PS and dissolve it in 1 ml [EMIM][TFSI] with 4 ml of anhydrous dichloromethane ($\mathrm{CH}_{2} \mathrm{Cl}_{2}$), and stir fully at 60℃. This can then be stored for 24 hours.

All samples were fabricated as per the following：

1. Surface treatment: Treat the organic residues on the surface with an ozone cleaner, then ultrasonically clean the substrate with acetone, ethanol, and deionized water, before drying.
2. Photoresist spin coating: Spin-coat a layer of photoresist on the substrate using a homogenizer. Generally, the glue spreads out at a low speed for a few seconds, and then becomes uniform under the action of centrifugal force caused by high-speed operation and will then cover the surface of the substrate.
3. Pre-bake: After the photoresist is spin-coated, baking is required to cure the liquid photoresist.
4. Exposure: By using a plate making machine, the pattern is exposed point by point on the photoresist coated substrate surface by direct laser writing.
5. Development: Wash and fix with deionized water, then dry with nitrogen to complete the pattern transfer.
6. Thin film deposition: A thin metal film is deposited on the developed substrate via electron beam evaporation.
7. Peeling: Use acetone to peel off the metal film (non-structured area) from the surface, then wash the sample with ethanol and deionized water alternately, and blow dry.


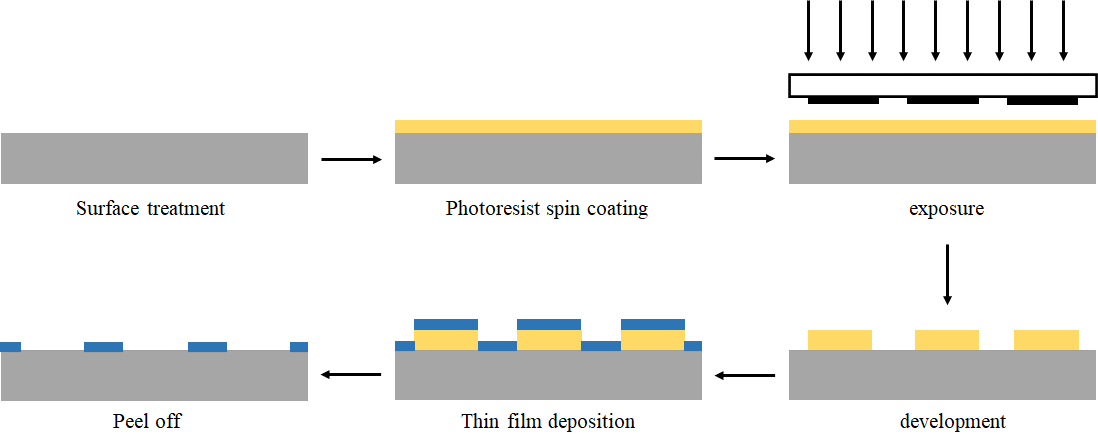


**Figure S3:** Process of sample fabrication.


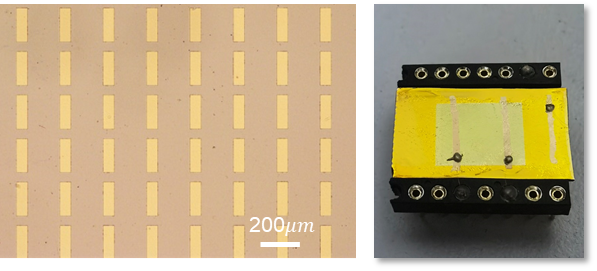


**Figure S4:** Sample images.

1. **Experimental characterization**

**3.1 Ion-gel gating of the graphene metasurface**

We employed an ion-gel gating technique to modulate the charge carrier density in graphene metasurfaces. The device configuration is schematically shown in Figure S5(a), where “T” is a bias connected to the ion-gel and “S” is the gate connected to the graphene layer, while “D” and “S” also construct drain-source base to measure drain-source current. The gate-dependent resistance of CVD-grown graphene transferred to the metasurface, as shown in Figure S5(b). The optical image of graphene samples is shown in the inset of Figure S5(b).


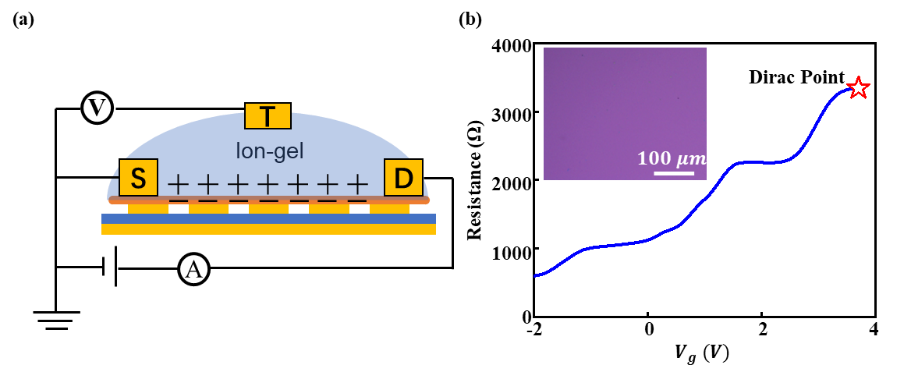


**Figure S5:** (a) Schematics of the ion-gel gating setup. (b) The gate-dependent resistance of CVD-grown graphene transferred to the metasurface. Inset: the optical image of graphene samples.

**3.2 Characterization of meta-atoms**

During the experiment, we used a THz time-domain spectroscopy (THz-TDS, see Figure S6) system to measure the graphene-integrated metasurface performance. By illuminating the samples via a horizontal polarization wave, setting the crystal axis of the samples to form an angle of 45° to the incident polarization angle, and rotating the polarizer in front of the receiver, we obtained the time-domain reflection signals along the *u/v* directions (see Figure S7(a)). Through Fourier transformation, we can obtain the frequency-domain signals along the *u/v* directions (see Figure S7(b)). For the LCP incidence, the normalized reflection intensity for abnormal and normal modes can be calculated as:

$\left\{\begin{matrix} \left|A_{a}\right|^{2}=\left|\frac{r_{uu}}{r_{0}}-\frac{r_{vv}}{r_{0}}\right|^{2}/\left(2\left|\frac{r_{uu}}{r_{0}}\right|^{2}+2\left|\frac{r_{vv}}{r_{0}}\right|^{2}\right)=\frac{\left|r_{uu}-r_{vv}\right|^{2}}{2\left(\left|r_{uu}\right|^{2}+\left|r_{vv}\right|^{2}\right)} \\ \left|A_{n}\right|^{2}=1-\left|A_{a}\right|^{2} \end{matrix}\right.$, (S12)

where $r_{uu}$ and $r_{vv}$ are the measured reflection coefficients along the *u* and *v* directions, and $r_{0}$ is the normalized energy. However, we must point out that during the measurement, it is not easy to simultaneously measure the $r_{uu}$, $r_{vv}$ and $r_{0}$ in the same architecture, we thus only employ the intensity for abnormal and normal modes $\left|A_{a/n}\right|^{2}$ in the main text. Figure S8, Figure S9 and Figure S10 plot the measured $r_{uu}$, $r_{vv}$ and $\left|A_{a/n}\right|^{2}$, respectively.


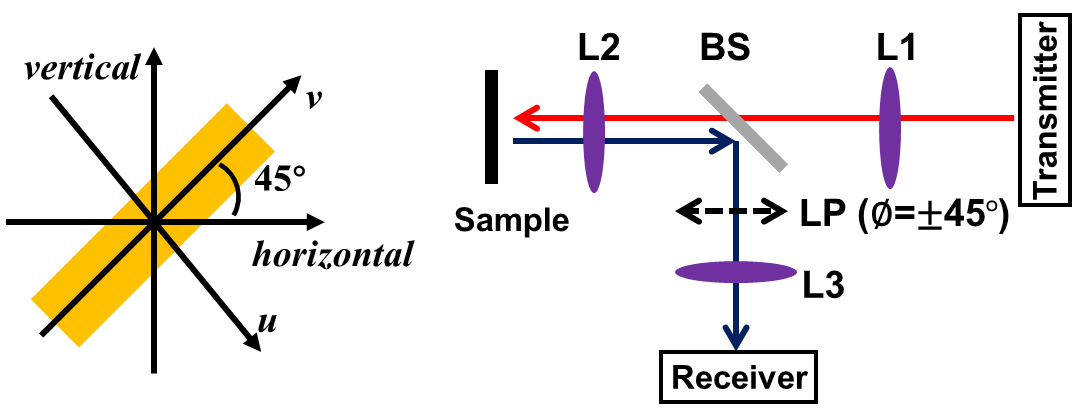


**Figure S6:** Schematic of the experimental setup to characterize the metasurface.


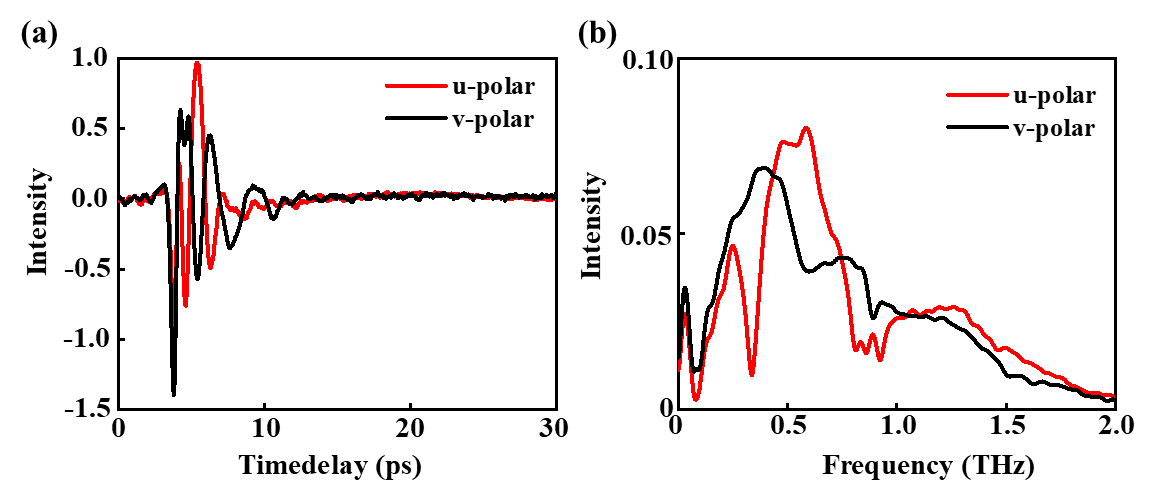


**Figure S7:** (a) Measured time-domain reflection signals along the *u* and *v* directions and (b) frequency-domain reflection signals along the *u* and *v* directions obtained by Fourier transformation at $V_{ext}=0 V$.


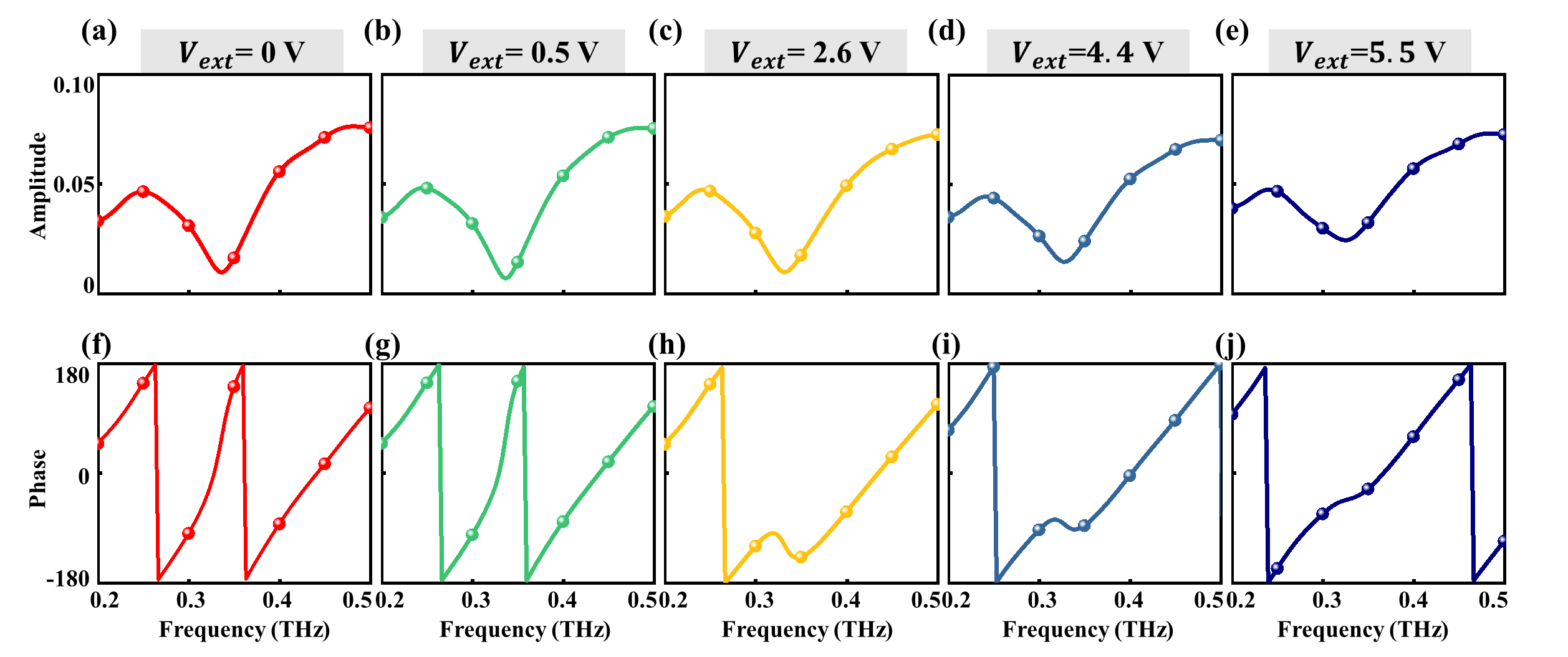


**Figure S8:** Measured amplitude and phase of $r_{uu}$ at five different gate voltages.


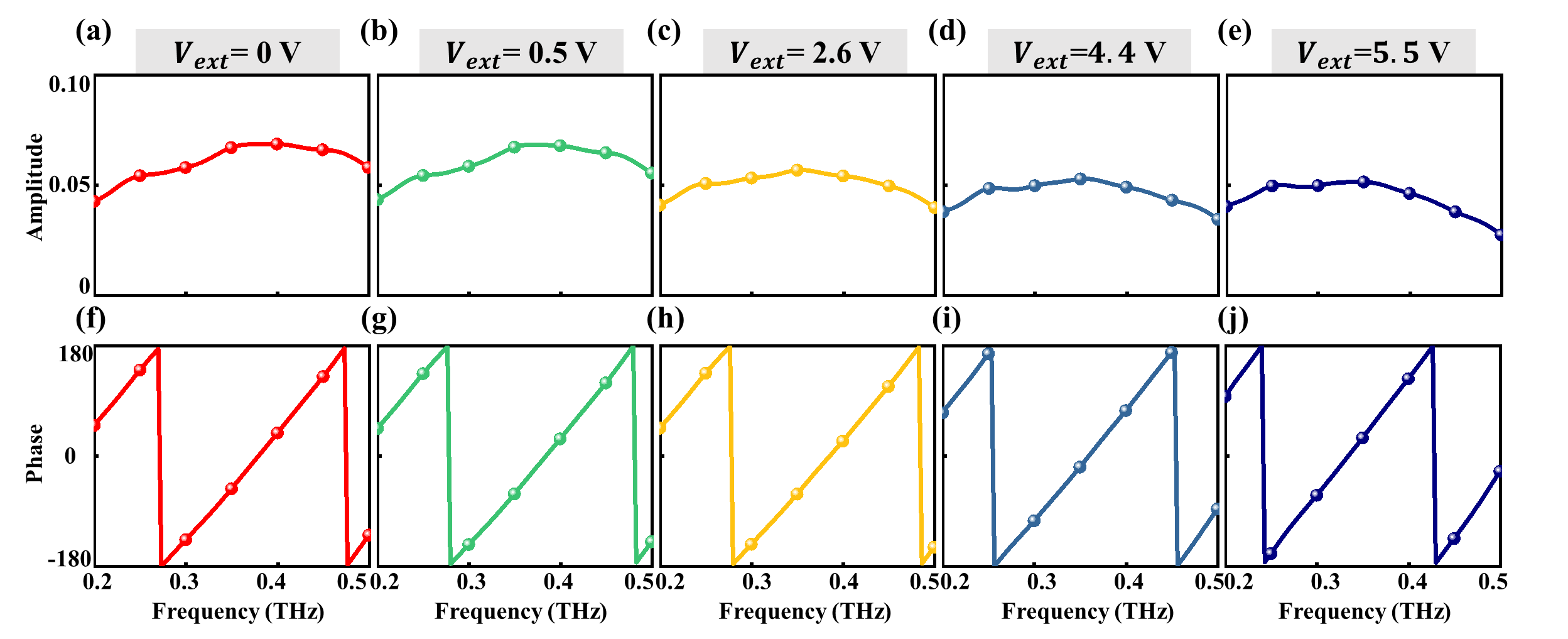


**Figure S9:** Measured amplitude and phase of $r_{vv}$ at five different gate voltages.


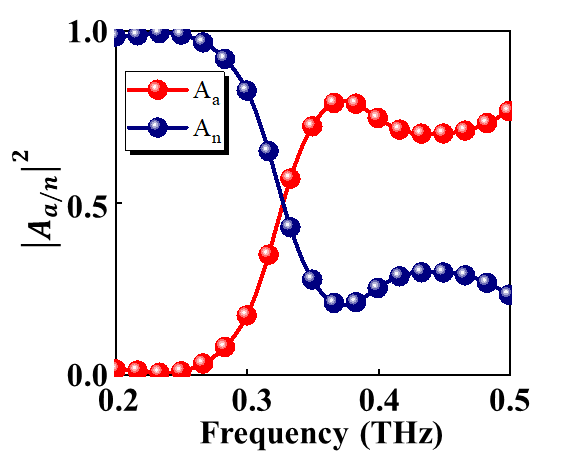


**Figure S10:** Normalized reflection intensities for normal and abnormal modes at$V_{ext}=0 V$.

**3.3 Characterization of the far-field scattering fields**

The signals emitted by the THz antenna are linear polarizations (*x* and *y* polarizations), where the four reflection signals $E_{xx}$, $E_{yx}$, $E_{xy}$, and $E_{yy}$ can be obtained through rotating the receiving antenna. Here, the first subscript represents the incident signals, and the second represents the received signals. We then rewrite these four reflection coefficients into a Jones matrix $J=\left(\begin{matrix} E_{xx} & E_{xy} \\ E_{yx} & E_{yy} \end{matrix}\right)$, and express it in CP bases as: $\tilde{J}=\frac{1}{2}\left(\begin{matrix} E_{xx}\text{+}E_{yy}+i(E_{xy}-E_{yx}) & E_{xx}-E_{yy}+i(E_{xy}-E_{yx}) \\ E_{xx}-E_{yy}+i(E_{xy}-E_{yx}) & E_{xx}+E_{yy}+i(E_{xy}-E_{yx}) \end{matrix}\right)$. Therefore, for LCP incidence, two signals for LCP and RCP can be obtained as $E_{RL}=[E_{xx}-E_{yy}+i(E_{xy}-E_{yx})]/2$, $E_{LL}=[E_{xx}\text{+}E_{yy}+i(E_{xy}-E_{yx})]/2$, as shown in Figure S11.


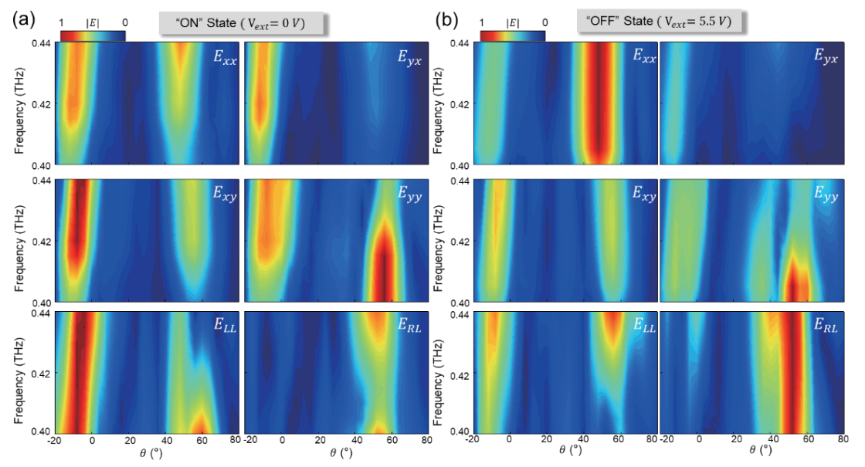


**Figure S11:** The measured angular distribution of FF scattered electric field intensities of reflected waves for (b) “ON” state with $V_{ext}=0 V$ and (c) “OFF” state with $V_{ext}=V_{max}$.

1. **Detailed information for full-wave simulations**

**4.1 Simulations of meta-atoms**

We performed numerical simulations using the commercial finite element method (FEM) solver CST Microwave Studio. In the numerical simulations, the conductivity of graphene in the THz regime can be described by the linear-response theory under random phase approximation, which is comprised of only intra-band contributions [1]:

$\sigma\left( \omega\right)\approx\frac{{ie}^{2}v_{f}\sqrt{n}}{\hbar\sqrt{\pi}(\omega+i\Gamma)}$, (S13)

where $v_{f}$ is the Fermi velocity of electrons in graphene (approximately $9\times{10}^{5} m/s$) [1], *n* is the two-dimensional electron density of graphene, and Γ is a phenomenological constant accounting for the electron scattering rate (damping). In the FDTD simulations, graphene was modeled as a thin dielectric layer with thickness d and with an anisotropic dielectric function [1] given by , where

$\varepsilon_{r}^{||}=\sqrt{\varepsilon_{r1}\varepsilon_{r2}}-\frac{\sigma\left( \omega\right)}{i\varepsilon_{0}\omega d}$ (S14)

is the standard Drude form. Here, $\varepsilon_{r1}$and $\varepsilon_{r2}$ are dielectric constants of two media surrounding the graphene and *d* is set as 30 nm. Notably, *d* is much larger than the real thickness of graphene, 0.3 nm. However, it was shown in Ref. [1] that the exact value of *d* does not influence the final result as long as *d* is considerably smaller than the wavelength, which was the case in our simulations. We assume that n is related to the gate voltage $V_{ext}$ through $N={(n_{0}^{2}+\alpha^{2}\left| V_{ext} \right|^{2})}^{1/2}$, where $\alpha$ is the gate capacitance in the ion-gel-based gating scheme and $n_{0}$ is the residual charge concentration, determined by fitting experimental data. In the simulations, optimal results were obtained for the following parameters: $n_{0}=1.84\times{10}^{15}m^{-2}$ and $\alpha={9.02}^{15}\times{10}^{15}m^{-2}V^{-1}$ for the Sec. III case, $n_{0}=1.18\times{10}^{15}m^{-2}$ and $\alpha={9.03}^{15}\times{10}^{15}m^{-2}V^{-1}$ for the Sec. IV case. Through substituting Sec. III case values into simulations, we obtained the meta-atom simulation results, which show good agreement with the experimental results, as demonstrated in Figure S12. In addition, we performed simulations to calculate the reflected Re(**E**) distributions in *xoz* plane for four representative cases $V_{ext}=0V$, $V_{ext}=0.55V$ $V_{ext}=0.71V$, and $V_{ext}=5.5V$, at the vicinity of the working frequency 0.38 THz, as shown in Figure S13. Clearly, with the increase of $V_{ext}$, the reflection phase for the $E||\hat{v}$ is almost unchanged, and the reflection phase for the $E||\hat{u}$ can be modulated from 0 to $\pi$.


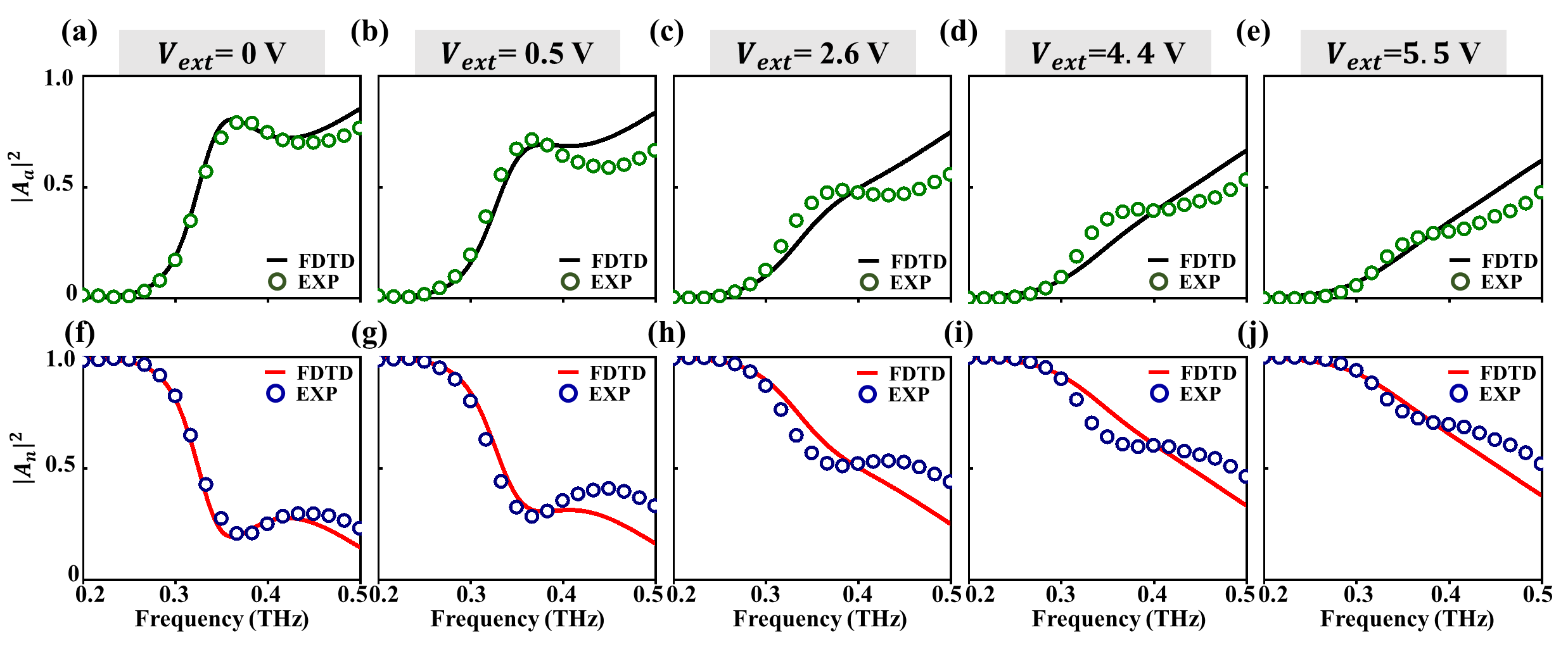


**Figure S12:** The intensity of (a-e) $\left| A_{a} \right|^{2}$ and (f-j) $\left| A_{n} \right|^{2}$ of the meta-atoms when different voltages are applied.


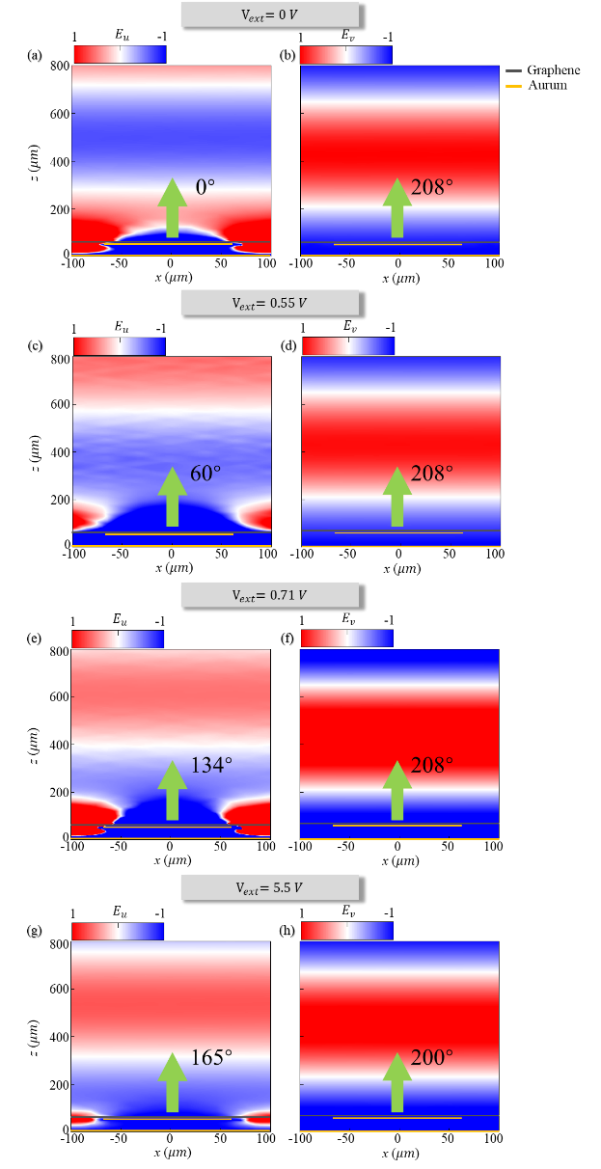


**Figure S13:** The simulated real part of electric field distributions for the reflected $E||\hat{u}$ and $E||\hat{v}$ polarizations in *xoz* plane for three representative cases (a,b) $V_{ext}=0V$, (c,d) $V_{ext}=0.55V$, (e,f) $V_{ext}=0.71V$, and (g,h) $V_{ext}=5.5V$.

**4.2 Simulation of the meta-deflector in Sec. IV**

We performed numerical simulations using the commercial Finite difference time domain method (FDTD) solver CST Microwave Studio. The structure of the meta-device in the simulation is shown in Figures S14(a,b). The meta-device is composed of $10\times32$ meta-atoms. The length of the metal patch is $l=140 um$, the width $w=40 um$, and the period $p= 200 um$. The simulation boundary conditions are set as absorption boundary conditions, the frequency is set as 0.42 THz, and the incident wave is set as a plane wave. The front view of the simulated array with normal and 50° incidence is shown in Figures S14(c,d), respectively. The 50° incidence simulation result is shown in Figure S15, where the far-field coordinate system was selected as the Ludwig 2 Azimuth over the Elevation coordinate system usually used in CST.


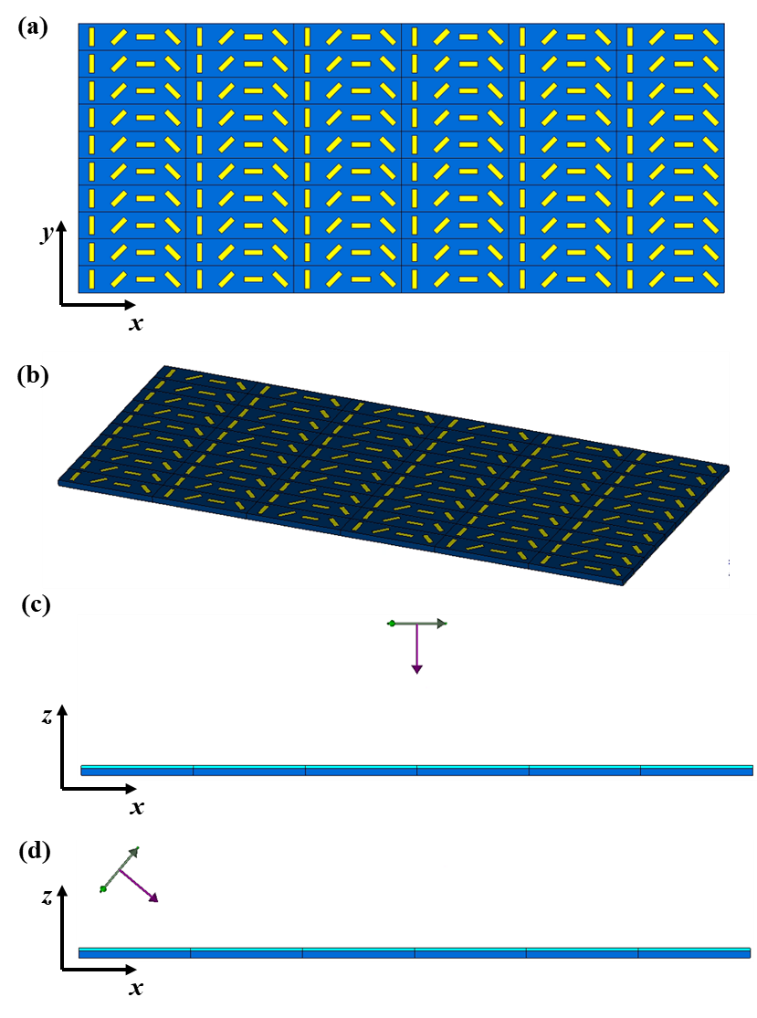


**Figure S14:** (a,b) The structure of the meta-device in simulation. A front view of the simulated array with (c) normal incidence and (d) 50° incidence.


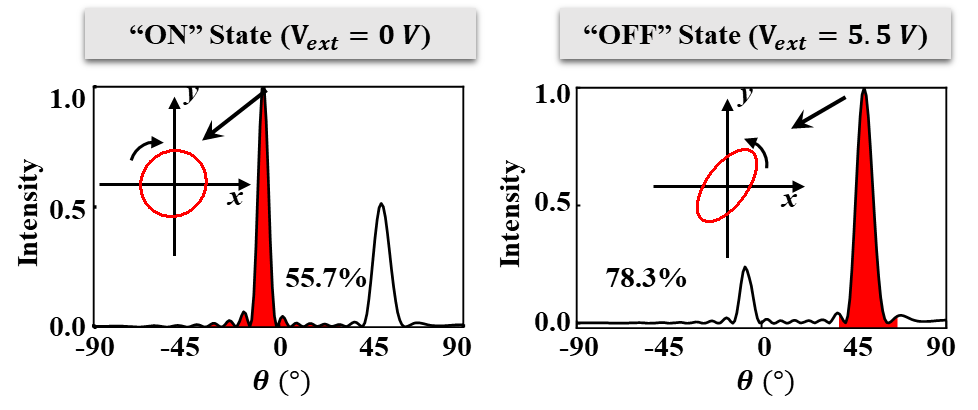


**Figure S15:** Simulated angular distribution of scattered electric field intensities, and the deflection efficiencies and polarizations when the meta-deflector is at “ON” state and “OFF” state with 50° incidence.

**4.3 Simulation of the meta-device in Sec. V**

We performed numerical simulations using the commercial Finite difference time domain method (FDTD) solver CST Microwave Studio. The structure of the meta-device in the simulation is shown in Figures S16(a,b). The meta-device is composed of $20\times20$ meta-atoms. The simulation boundary conditions are set as absorption boundary conditions, the frequency is set as 0.42 THz, and the incident wave is an LCP Laguerre-Gaussian beam with $E^{in}\left(r\right)\text{=}\exp\left[-(x^{2}+y^{2})/w_{0}^{2}\right]\exp\left[-i\arg\left(x+iy\right)\right]$.


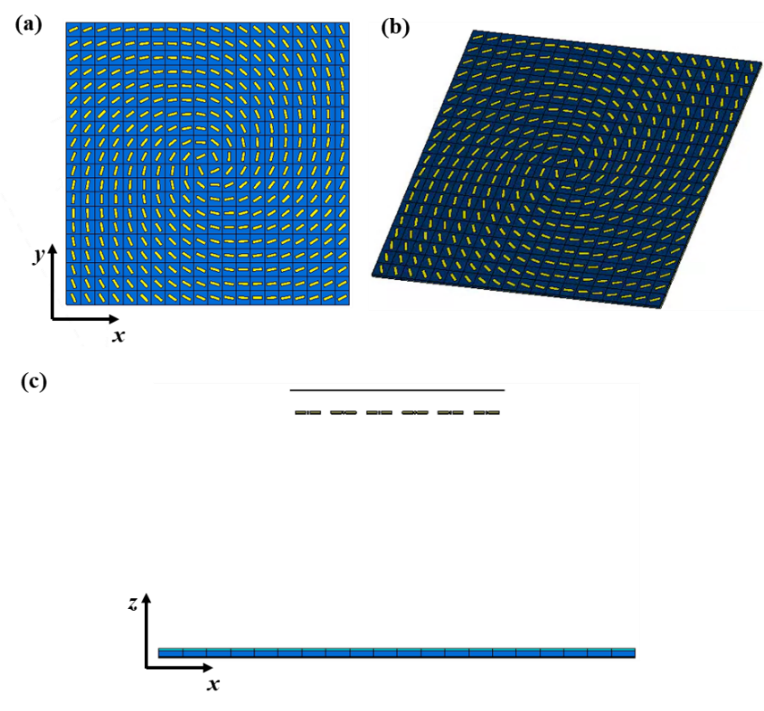


**Figure S16:** (a,b) The structure of the meta-device in simulation. (c)The front view of the simulated array with normal incidence.

1. **CMT fitting for meta-atoms**

We performed the FDTD simulations of meta-atoms, as described in the main text, and fitted the simulation results with the theoretical results obtained by CMT. Table S1 shows the fitting results of some parameters. Figure S17 and Figure S18 depict the fitting spectral lines of amplitudes and phase along the *u* and *v* directions of the meta-atoms when different voltages are applied, Figure S19 and Figures S20 depict the fitting spectral lines of intensity and phase for abnormal and normal modes when different voltages are applied.

**Table S1** CMT parameters.

| $V_{ext}$ (V) | $\Gamma_{1}$ (THz) | $\Gamma_{1}^{A}$ (THz) | $f_{1}$ (THz) | $C_{1}$ | $\Gamma_{2}$ (THz) | $\Gamma_{2}^{A}$ (THz) | $f_{2}$ (THz) | $C_{2}$ |
| --- | --- | --- | --- | --- | --- | --- | --- | --- |
| 0 | 0.04 | 0.03 | 0.34 | $e^{-i0.79}$ | 0.14 | 0.02 | 0.56 | $e^{-i0.44}$ |
| 0.5 | 0.04 | 0.04 | 0.34 | $e^{-i0.79}$ | 0.14 | 0.03 | 0.56 | $e^{-i0.44}$ |
| 2.6 | 0.04 | 0.08 | 0.34 | $e^{-i0.79}$ | 0.14 | 0.06 | 0.57 | $e^{-i0.44}$ |
| 4.4 | 0.04 | 0.10 | 0.34 | $e^{-i0.79}$ | 0.14 | 0.08 | 0.57 | $e^{-i0.44}$ |
| 5.5 | 0.04 | 0.11 | 0.34 | $e^{-i0.79}$ | 0.14 | 0.09 | 0.57 | $e^{-i0.44}$ |


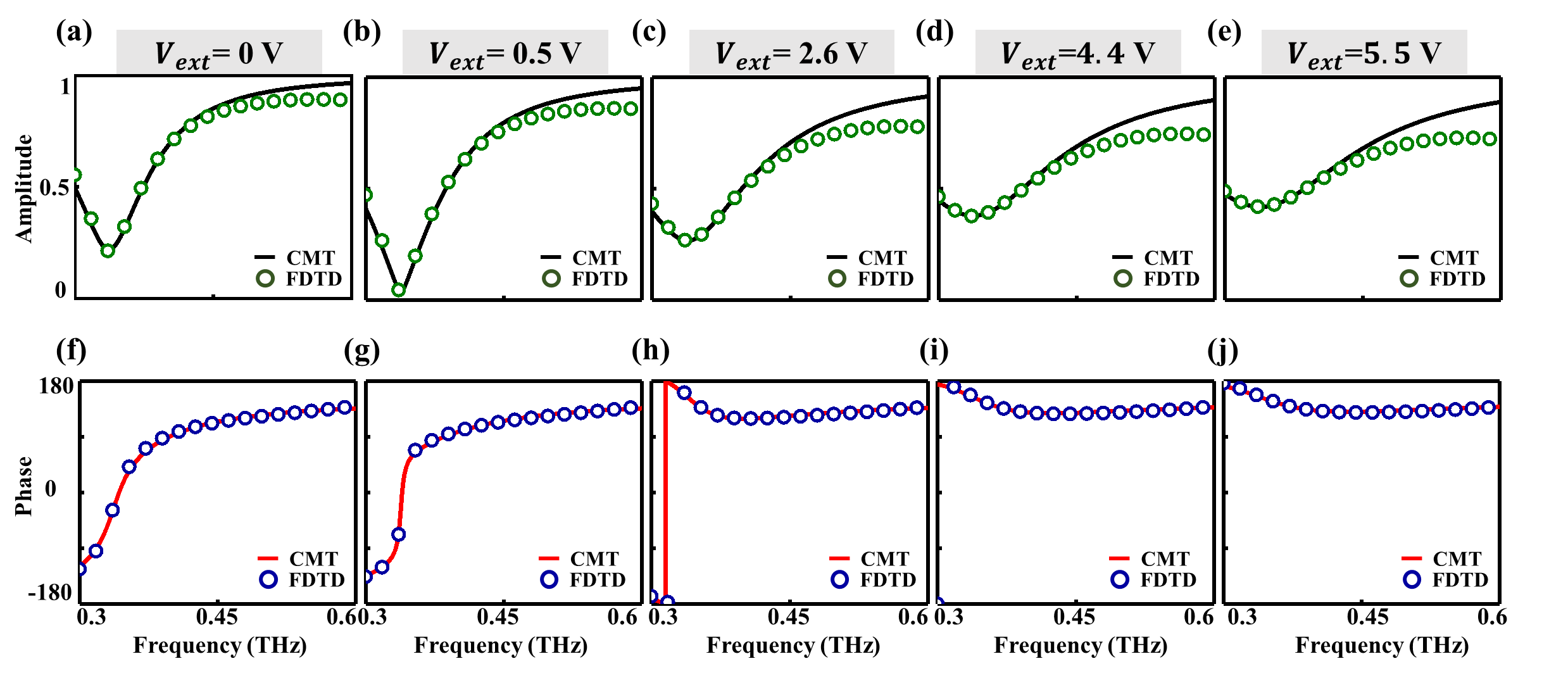


**Figure S17:** The fitting spectral lines of (a-e) amplitudes and (f-j) phase along the *u* direction of the meta-atoms when different voltages are applied.


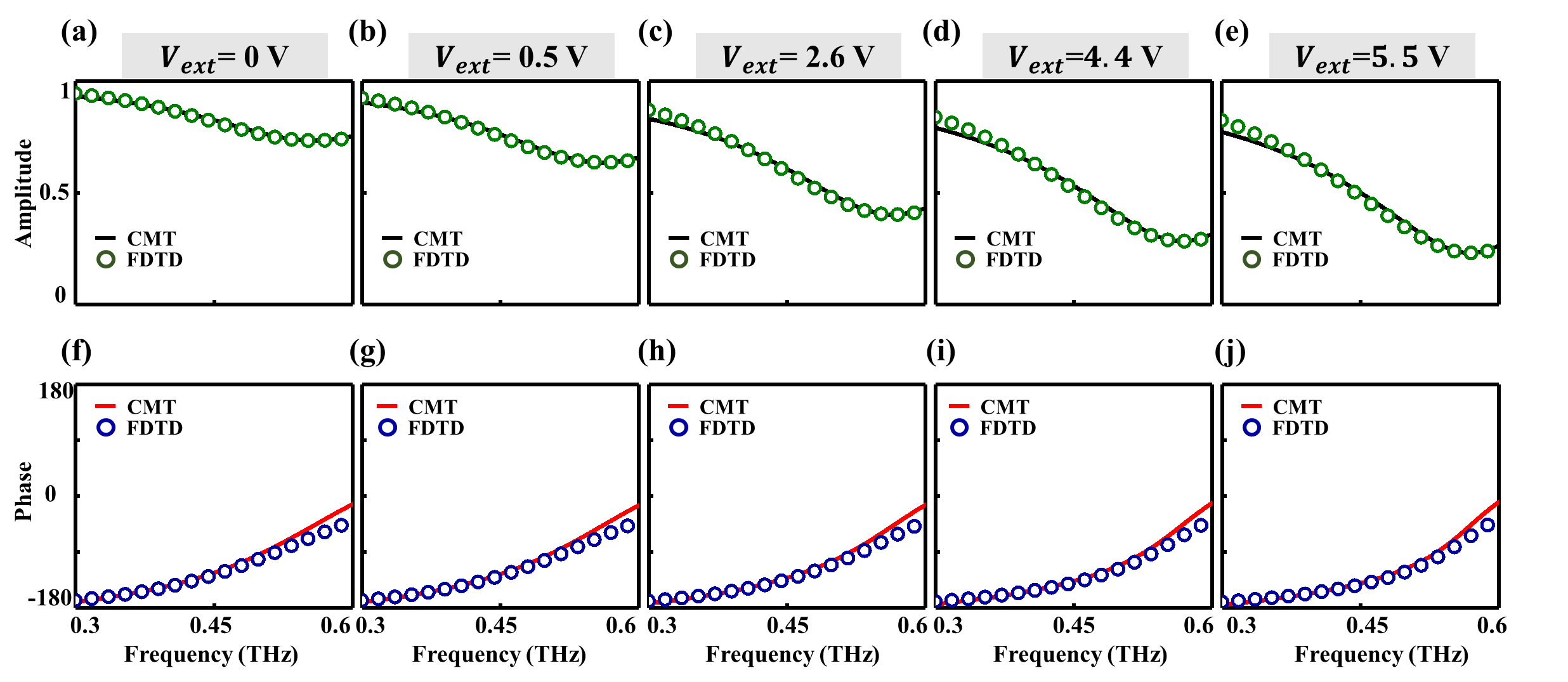


**Figure S18:** The fitting spectral lines of (a-e) amplitudes and (f-j) phase along the *v* direction of the meta-atoms when different voltages are applied.


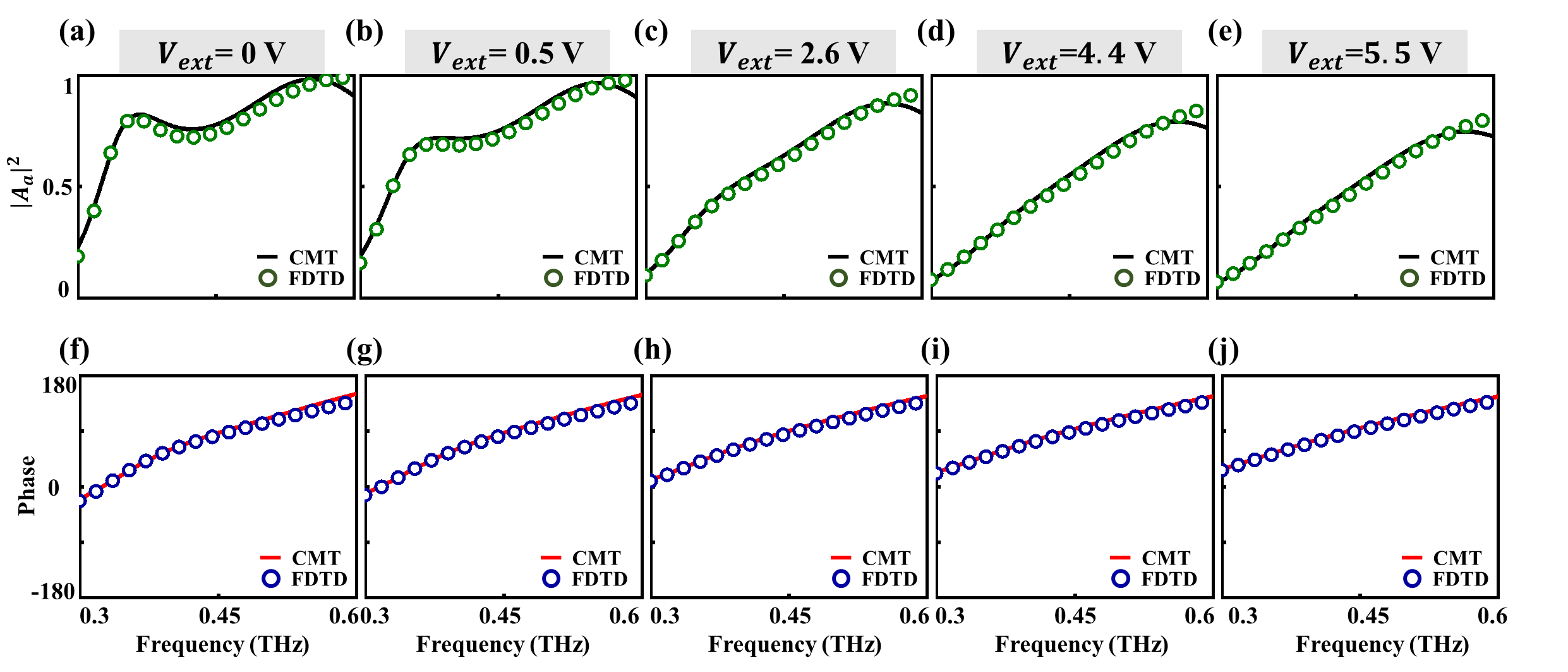


**Figure S19:** The fitting spectral lines (a-e) of abnormal mode intensity and (f-j) phase when different voltages are applied.


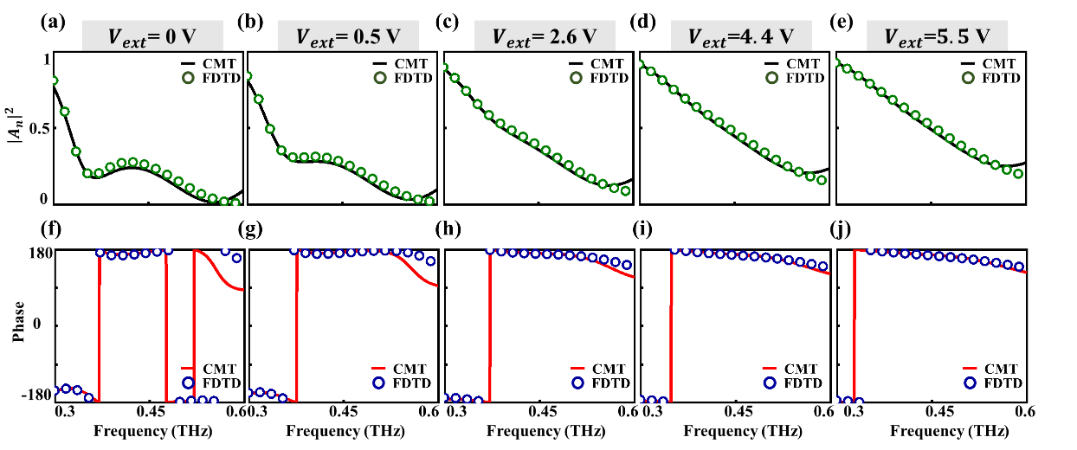


**Figure S20:** The fitting spectral lines of (a-e) normal mode intensity and (f-j) phase when different voltages are applied.

1. **Possible methods for further improving the efficiency**

Due to the existence of the ohmic losses of material (e.g. the Au metal, ion-gel, and the spacer). In our experiment, our fabricated sample module the graphene carrier concentration from $1.18\times10^{15}m^{-2}$ to $2.12\times10^{16}m^{-2}$, and therefore exhibit modulation efficiency around 60%. However, it is worth to mention that such efficiency can be further enhanced by use of better architecture, i.e. back-gate, and improved-quality graphene. We performed a simulation based on the settings of back-gate in the Ref. [3], with improved variation range of graphene carrier concentration as ($9.07\times10^{13}m^{-2}\sim$ $1.11\times10^{17}m^{-2}$) according to the Ref. [3]. The back-gated meta-device is schematically shown in Figure S21. A graphene/Al_2_O_3_/TiO_x_ sandwich structure is constructed for a solid-state electrical gate to tune the conductivity of graphene. A 50-nm Al_2_O_3_ layer is used as a dielectric gate, and the back gate electrode is made of a 10-nm TiO_x_ film. The length of the metal patch is$l=158 \mu m$, the width$w=45 \mu m$, and the period$p=200 \mu m$. The simulation boundary conditions are set as absorption boundary conditions, the frequency is set as 0.42 THz, and the incident wave is set as a plane wave. Full-wave simulation illustrate that the efficiency of the graphene meta-device can be improved to around 80% (see Figure S21).


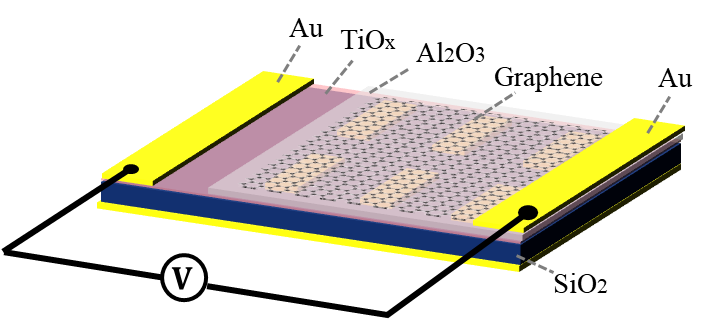


**Figure S21:** The Schematic of back-gated meta-device.

The efficiency of the device can be improved to about 80%, the simulation results are shown in Figure S22.


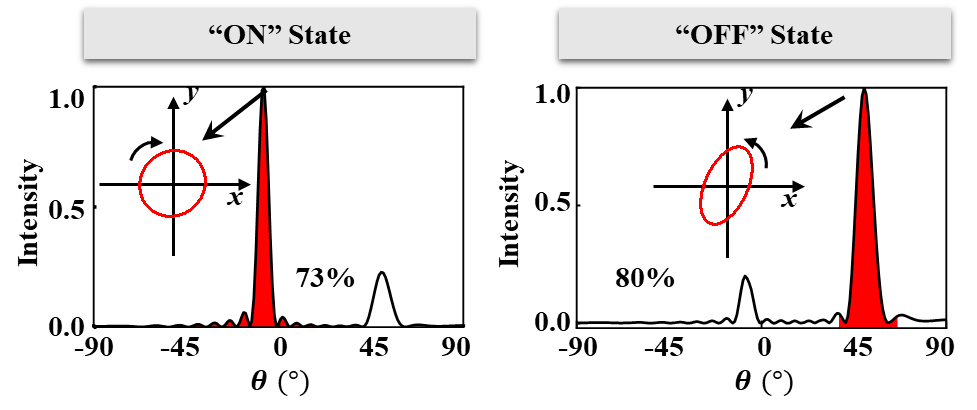


**Figure S22:** The deflection efficiencies and the scattered polarizations under two different gate voltages $V_{ext}$= 0V (“ON” state) and $V_{ext}=V_{max}$ (“OFF” state).

1. **Flexible wavefront control, based on resonance and PB phases**

We subsequently designed a meta-device with a geometric phase $\Phi_{geo}=-0.9k_{0}x$ and a resonance phase $\Phi_{res}=0.6k_{0}x$. After illuminating such a meta-device with a normal LCP incident plane wave ($E^{in}\left(r\right)=1$), we calculated the simplified theoretical form for the total scattering electric field as:

$E^{ref}\left(k;V_{ext}\right)=\left(\begin{matrix} A_{a}\left(V_{ext}\right)\left[\delta\left(k_{x}+0.3k_{0}\right)\delta\left(k_{y}\right)\right] \\ A_{n}\left(V_{ext}\right)\left[\delta\left(k_{x}-0.6k_{0}\right)\delta\left(k_{y}\right)\right] \end{matrix}\right)$, (S15)

indicating that there are two anomalous reflected waves in the scattering field with:

$\left\{\begin{matrix} \theta_{a}=-17.5^{\circ} \\ \theta_{n}=37^{\circ} \end{matrix}\right.$. (S16)

Based on Eq. (S7), we calculated the angular distribution of the scattered electric field intensities, the deflection efficiencies, and polarizations at $A_{a}\left(0\right)>>A_{n}\left(0\right)$ (“ON” state), $A_{a}\left(V_{ext}\right)=A_{n}\left(V_{ext}\right)$ (“MIX” state) and $A_{a}\left(V_{max}\right)<<A_{n}\left(V_{max}\right)$ (“OFF” state), as shown in Figure S23.


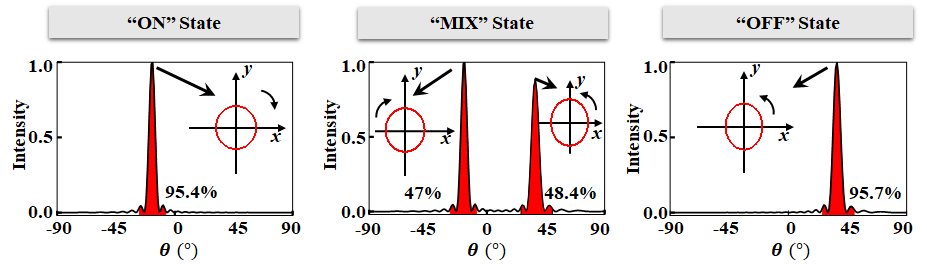


**Figure S23:** Theoretical angular distribution of the scattered electric field intensities, the deflection efficiencies and polarizations when the meta-deflector is at “ON” state, “MIX” state, and “OFF” state, after introducing the geometric and resonance phases

**References**

1. K. Ding, Y. Shen, J. Ng, and L. Zhou, “Equivalent-Medium Theory for Metamaterials Made by Planar Electronic Materials,” *Europhys. Lett.* **102**(2), 28005 (2013).
2. J. Lin, M. Qiu, X. Zhang, H. Guo, Q. Cai, S. Xiao, Q. He, and L. Zhou, “Tailoring the lineshapes of coupled plasmonic systems based on a theory derived from first principles,” *Light: Sci. Appl.* **9**, 158 (2020).
3. Z. Chen, X. Chen, L. Tao, K. Chen, M. Long, X. Liu, K. Yan, R. I. Stantchev, E. Pickwell-MacPherson and J. Xu, “Graphene controlled Brewster angle device for ultra broadband terahertz modulation,” *Nat. Commun.* 9(1), 4909 (2018).
